# Supplementary material for: An Arginine-Rich Motif in the ORF2 capsid protein regulates the hepatitis E virus lifecycle and interactions with the host cell
Source: PLoS Pathog. 2022 Aug 25;18(8):e1010798. doi: 10.1371/journal.ppat.1010798 (PMC9451086; doi:10.1371/journal.ppat.1010798)
Supplement: S1 Text — (DOCX) [file ppat.1010798.s001.docx]

**S1 Text**

**An Arginine-Rich Motif in the ORF2 Capsid Protein Regulates the Hepatitis E Virus Lifecycle and Interactions with the Host Cell**

Kévin Hervouet,^1,¶^ Martin Ferrié,^1,¶^ Maliki Ankavay,^1,2,¶^ Claire Montpellier,^1^ Charline Camuzet,^1^ Virginie Alexandre,^1^ Aïcha Dembélé,^1^ Cécile Lecoeur,^1^ Arnold Thomas Foe,^1^ Peggy Bouquet,^3^ David Hot,^3^ Thibaut Vausselin,^1^ Jean-Michel Saliou,^3^ Sophie Salomé-Desnoulez,^3^ Alexandre Vandeputte,^3^ Laurent Marsollier^4^, Priscille Brodin,^1,3^ Marlène Dreux,^5^ Yves Rouillé,^1^ Jean Dubuisson,^1^ Cécile-Marie Aliouat-Denis,^1^ Laurence Cocquerel,^1,^*

**Legends of supporting Figures**

**S1 Fig. Conservation of the Arginine-Rich Motif (ARM) in the ORF2 sequence.** Fifty ORF2 amino acid (aa) sequences representative of the four mostly prevalent human HEV genotypes were aligned using Clustal Omega program. The ARM corresponding to aa 28-33 in the p6 strain is boxed in red.

**S2 Fig. Effect of 5R/5A, PSG/3R and ΔSP mutations on the kinetics of ORF2 subcellular localization.** PLC3 cells were electroporated with ARM/SP mutant RNA and fixed at the indicated timepoints post-electroporation (p.e.). Indirect immunofluorescence analysis was performed using the 1E6 anti-ORF2 antibody. Cells were analyzed by confocal microscopy (magnification x40). Scale bar, 20 μm. Nuclear/cytosolic fluorescence intensity quantification was done using ImageJ software (mean ± S.D., *n* ≥ 30 cells, Friedman with Nemenyi test). Groups at 18 h.p.e. and 72h.p.e. were compared with a t-test. **p < 0.01, ***p < 0.001, and *********p* < 0.0001.

**S3 Fig. Colocalization analysis of ORF2 with the Golgi marker 130 (GM130) in PLC3/HEV-p6 cells expressing ORF2wt or ARM/SP mutants.** PLC3/HEV-p6 cells expressing ORF2wt or ARM/SP mutants were analyzed by indirect immunofluorescence at 18 h.p.e. Cells were analyzed by confocal microscopy (magnification x63). Representative images are shown. Scale bar, 20 μm. Pearson’s correlation coefficients were calculated using JACoP plugin from ImageJ software using the whole cell as ROI (mean ± S.D., *n* ≥ 30 cells, Kruskal-Wallis with Conover’s test). ********p* < 0.001, *********p* < 0.0001. Red = ORF2; Green = GM130; Blue = DAPI.

**S4 Fig. Controls of RNA and infectious titers.** Infectious titer determination and HEV RNA quantification in PLC3/HEV-p6 expressing ORF2wt, 5R/5A or NES12 mutant proteins, in PLC3 cells electroporated with the replication-defective p6 GAD mutant and in mock electroporated PLC3 cells. At 7 d.p.e, PLC3/HEV-p6 cells were also treated with 20 μM of Sofosbuvir or diluent (DMSO) for 72h. (A, B) Extra- and intracellular viral particles were extracted at 10 d.p.e and used to infect naïve Huh7.5 cells for 3 days. Cells were next processed for indirect immunofluorescence. ORF2-positive cells were counted and each positive cell focus was considered as one FFU. Results were expressed in FFU/ml (*n*=3). (C, D) Extra- and intracellular viral RNAs were quantified at 10 d.p.e by RT-qPCR (*n*=3) (mean ± S.D., Kruskal-Wallis with Conover’s test). ******p* < 0.05, *******p* < 0.01, ********p* < 0.001, *********p* < 0.0001.

**S5 Fig. Colocalization analysis of ORF2 with the Importin-α1 in PLC3/HEV-p6 cells expressing ORF2wt and ARM/SP mutants.** PLC3/HEV-p6 cells expressing ORF2wt or ARM/SP mutants were analyzed by indirect immunofluorescence at 18 h.p.e. Cells were analyzed by confocal microscopy (magnification x63). Representative images are shown. Scale bar, 20 μm. Pearson’s correlation coefficients were calculated using JACoP plugin from ImageJ software using the whole cell as ROI (mean ± S.D., *n* ≥ 30 cells, Kruskal-Wallis with Conover’s test). *********p* < 0.0001. Red = ORF2; Green = Importin-α1; Blue = DAPI.

**S6 Fig. ORF2 nuclear translocation modulates host gene expression.** (A) Transcriptomic analysis of PLC3 cells expressing HEV-p6 wt, mutants or mock cells performed with microarrays (Agilent SurePrint). Left - Heatmap of gene expression in PLC3 cells expressing HEV-p6 wt, mutants or mock cells at 18 h.p.e. Color-code represents the log fold-change (logFC) of gene expression in the indicated comparisons. Middle – STRING database (<https://string-db.org>) representation of the protein-protein interaction network of the genes modulated by ORF2. Right – Signaling pathways preferentially induced by the nuclear translocation of ORF2 identified through Gene Ontology enrichment using Metascape (<https://metascape.org>). Transcriptomic results stem from 4 independent electroporation experiments. (B) Representative images of ORF2 subcellular localization in PLC3/HEV-p6 cells expressing ORF2wt, 5R/5A mutant, ΔORF3 mutant or Mock-electroporated PLC3 cells at 18 h.p.e, the time point at which RNA extraction and transcriptomic analyses were performed. Cells were analyzed by confocal microscopy (magnification x63). Red = ORF2; Blue = DAPI. Scale bar, 20μm. (C) Representative images showing the ORF3 expression in cells used for transcriptomic analyses. Since ORF3 is poorly expressed at early time points post-electroporation, electroporated cells were fixed at 6 d.p.e. and processed for ORF2 and ORF3 staining. Cells were analyzed by confocal microscopy (magnification x63). Red = ORF2; Green = ORF3; Blue = DAPI. Scale bar, 20μm. (D) Intracellular RNAs were quantified at 18 h.p.e by RT-qPCR using CCL2, CCL20, CXCL1, CXCL2, NFKBIA, TNFAIP2 or TNFAIP3-targeting probes. Intracellular HEV RNA levels were controlled using an ORF1-targeting probe. *n* ≥ 6, Kruskal-Wallis with Conover’s test for CCL2 and TNFAIP2 and ANOVA with Dunnett’s test for the other genes. ******p* < 0.05, *******p* < 0.01, *********p* < 0.0001.

**S7 Fig. Effect of ΔORF3 and NES12 mutations on the kinetics of ORF2 subcellular localization.** (A) Total extracts of PLC3 mock, PLC3/HEV-p6-wt, PLC3/HEV-p6-NES12, PLC3/HEV-p6-PSG/3R, PLC3/HEV-p6-5R/5A, and PLC3/HEV-p6-$\Delta$ORF3 cells were analyzed by WB with anti-ORF2 (1E6), anti-ORF3 and anti-tubulin antibodies. Molecular mass markers are indicated on the right (kDa). (B) and (C) PLC3 cells were electroporated with ΔORF3 or NES12 mutant RNA and fixed at the indicated timepoints p.e. Indirect immunofluorescence analysis was performed using the 1E6 anti-ORF2 antibody. Cells were analyzed by confocal microscopy (magnification x40). Scale bar, 20 μm. Nuclear/cytosolic fluorescence intensity quantification was done using ImageJ software (mean ± S.D., *n* ≥ 30 cells, Friedman with Nemenyi test). Groups at 18 h.p.e. and 72h.p.e. were compared with a t-test. *********p* < 0.0001.

**S8 Fig.** **Nuclear export, colocalization and interaction of ORF2 with the exportin CRM1 in cells expressing ORF2wt or NES mutants.** (A) Analysis of ORF2 nuclear export in inhibitors treated-PLC3/HEV83-2 and HEV-p6 infected Huh-7.5 cells. Cells were treated at 18 h.p.e and 5 days p.i., respectively, with 20nM of Leptomycin B (LepB), 100nM of Verdinexor (Verd) or diluent (EtOH or DMSO, respectively) for 16h. Cells were processed for indirect immunofluorescence with the 1E6 anti-ORF2 Ab and analyzed by confocal microscopy (magnification x63). Red = ORF2; Blue = DAPI. Nuclear/cytosolic fluorescence intensity quantification was done using ImageJ software (mean ± S.D., *n* = 30 cells, Mann-Whitney test). *********p* < 0.0001. (B) PLC3/HEV-p6 cells expressing ORF2wt or NES mutants were analyzed by indirect immunofluorescence at 48 h.p.e. Cells were analyzed by confocal microscopy (magnification x63). Representative images are shown. Red = ORF2; Green = CRM1; Blue = DAPI. Pearson’s correlation coefficients were calculated using JACoP plugin from ImageJ software using the whole cell as ROI (mean ± S.D., *n* ≥ 30 cells, Kruskal-Wallis with Conover’s test). ********p* < 0.001, *********p* < 0.0001. (C) PLC3/HEV-p6-wt, PLC3/HEV-p6-NES12, and PLC3 mock cells were processed for proximity ligation assay using antibodies to ORF2 and CRM1 at 48 h.p.e. Stacks of images corresponding to the total volume of the cells were acquired, and maximum intensity projections of the stacks were generated. For each condition, 12 fields of cells were analyzed (total cell number ≥ 169). Scaled regions of interest of a representative field (left) and quantification of spot/cell (right) are shown (mean ± S.D., Kruskal-Wallis with Dunn’s test). ******p* < 0.05, *********p* < 0.0001. Scale bar, 20 μm.

**S9 Fig. Conservation of the Nuclear Export Signal 9 (NES9) in the ORF2 sequence.** Fifty ORF2 aa sequences representative of the four mostly prevalent human HEV genotypes were aligned using Clustal Omega program. The NES9 corresponding to aa 465-475 in the p6 strain is boxed in red.

**S10 Fig. Conservation of the Nuclear Export Signal 10 (NES10) in the ORF2 sequence.** Fifty ORF2 aa sequences representative of the four mostly prevalent human HEV genotypes were aligned using Clustal Omega program. The NES10 corresponding to aa 539-547 in the p6 strain is boxed in red.

**S11 Fig. Conservation of the Nuclear Export Signal 12 (NES12) in the ORF2 sequence.** Fifty ORF2 aa sequences representative of the four mostly prevalent human HEV genotypes were aligned using Clustal Omega program. The NES12 corresponding to aa 640-650 in the p6 strain is boxed in red.

**S12 Fig.** **Addressing of C2 constructs.** Schematic representation of the ORF2wt protein. SP ORF2 residues are shown in blue. ARM residues are highlighted in red. (A) H7-T7-IZ cells were transfected with pTM plasmids expressing ORF2wt or C2 constructs. In these constructs, the first half of the ORF2 SP (SP1) was deleted. Twenty-four hours post-transfection, cells were fixed and processed for ORF2 staining (in red). Nuclei are in blue. Representative confocal images are shown together with ORF2/DAPI merge images. Blue dots observed in some pictures are DAPI-stained transfected plasmids. A schematic representation of each construct is shown on the left. Scale bar, 20μm. (B) Nuclear-to-cytoplasmic ORF2 staining ratio in H7-T7-IZ cells expressing ORF2wt or C2 constructs. Quantification was done using ImageJ software (mean ± S.D., *n* ≥ 30 cells, Kruskal-Wallis with Conover’s test). *******p* < 0.01, *********p* < 0.0001. (C) Subcellular fractionation of H7-T7-IZ cells expressing ORF2wt, C2 constructs or the pTM empty vector, at 24h post-transfection. Fractionation was done using the subcellular protein fractionation kit for cultured cells. ORF2 proteins were detected by WB with the 1E6 Ab. Tubulin, Calnexin (CNX) and Lamin B1 were also detected to control the quality of fractionation. Molecular mass markers are indicated on the right (kDa).

**S13 Fig. Dose-response curves of PLC3 cells treated with the different drugs used in this study.** Cell viability was determined by a MTS based assay. Cells treated with the solvent (DMSO or Ethanol) were used as a control and set to 100%. High concentrations of Gossypol were used as a cytotoxic control. For LepB and Verd, blue arrows indicate the concentration used for subsequent experiments. Times of treatment are indicated.

**S1 Data.** Excel spreadsheet containing, in separate sheets, the underlying numerical data and statistical analysis for Fig 1 and Figs 2B, 2D Top, 2D Bottom, 2E, 3A, 3C, 3E, 3F, 5B, 6B, S2, S3, S4, S5, S6A Left, S6A Right, S6D, S7, S8A, S8B, S8C, S12, and S13

**S2 Data.** Compressed file containing figures of the unprocessed gels used in figures 2C, 3D, 4A and C, 4D-F, 4G, 5C, 6D, S7A and S12C.

**Supporting Materials and Methods**

**Sequence alignment.** Fifty ORF2 amino acid sequences representative of the four mostly prevalent human HEV genotypes were downloaded from NCBI database (<https://www-ncbi-nlm-nih-gov.insb.bib.cnrs.fr/>). Multiple alignments of these sequences were conducted using the Clustal Omega program (<https://www.ebi.ac.uk/Tools/msa/clustalo/>).

**RNA extraction and quantification.** HEV RNA levels were quantified by RT-qPCR using primers (5’-GGTGGTTTCTGGGGTGAC-3’ (F) and 5’-AGGGGTTGGTTGGATGAA-3’ (R)) and a probe (5’-FAM-TGATTCTCAGCCCTTCGC-TAMRA-3’) that target a conserved 70 bp region in the ORF2/3 overlap. In **S6 Fig,** HEV RNA levels were quantified by RT-qPCR using primers (5’-: AAGACATTCTGCGCTTTGTT-3’ (F) and 5’- TGACTCCTCATAAGCATCGC-3’ (R)) and a probe (5’-FAM- CCGTGGTTCCGTGCCATTGA-TAMRA-3’) that target a conserved region of ORF1. HEV RNAs were extracted from culture supernatants with the QIAmp viral RNA mini kit (Qiagen) and from cells with the Nucleospin RNA Plus kit (Macherey & Nagel). Retrotranscription was performed using the AffinityScript Multiple temperature cDNA synthesis Kit (Agilent Technologies) according to manufacturer’s instructions. Amplifications were done with a Quant Studio 3 apparatus (Applied Biosystems) and Taqman universal master mix no AmpErase UNA (Applied Biosystems). Cellular gene RNA levels were quantified by RT-qPCR using in-home primers (see **S2 Table**) and standards. Total cellular RNAs were extracted using TRIzol (Invitrogen) according manufacturer’s instructions and processed for retrotranscription using the High-capacity reverse transcription kit (Applied Biosystems). Amplifications were done with a Quant Studio 3 apparatus (Applied Biosystems) and SYBRGreen PCR Master Mix (Applied Biosystems).

**Transcriptomic analysis.** PLC3 cells were electroporated with HEV-p6-wt, HEV-p6-5R/5A, HEV-p6-$\Delta$ORF3 RNAs or no RNA (mock). At 18 h.p.e. total cellular RNAs were extracted using TRIzol (Invitrogen) according manufacturer’s instructions. RNA integrity and purity were verified using the Agilent Bioanalyzer system (Agilent Technology). Two µg of total RNA were treated with 2 units of DNaseI (Sigma Aldrich) during 10 min before purification on Nucleomag NGS cleanup beads (Macherey Nagel). Oligonucleotide microarrays for human whole genome (G4858A design 072363, 8x60k chips SurePrint G3 unrestricted GE, Agilent Technologies) were used for global gene expression analysis. Two hundred ng of total RNA was used in the Agilent Quick-Amp Labeling kit according to manufacturer’s instructions. After purification using an RNeasy Mini Kit (Qiagen), cRNA yield and incorporation efficiency (specific activity) into the cRNA were determined using a NanoDrop 2000 (Thermo Scientific) spectrophotometer. For each sample, a total of 600 ng of cRNA was fragmented and hybridized overnight at 65°C. After hybridization, slides were washed before being scanned on a SureScan Microarray Scanner (Agilent Technologies) and further processed using Feature Extraction v10.7.3.1 software. The resulting text files were uploaded into language R v4.0.3 and analyzed using the LIMMA package (Linear Model for Microarray Data) [1,2]. A ‘within-array’ normalization was performed using LOWESS (locally weighted linear regression) to correct for dye and spatial effects [3]. Moderate *t*-statistic with empirical Bayes shrinkage of the standard errors [4] was then used to determine significantly modulated genes. Statistics were corrected for multiple testing using a false-discovery rate approach. Protein-protein interactions network was generated using STRING database [5]. Gene ontology enrichment was performed using Metascape resource [6] ([www.metascape.org](http://www.metascape.org)) on the significantly modulated genes to identify pathways significantly modulated by either wild-type or mutants.

**Supporting references**

1. Brownstein MJ, Khodursky A, Smyth GK, Yang YH, Speed T. Functional Genomics, Methods and Protocols. Methods Mol Biology Clifton N J. 2003;224: 111–136. doi:10.1385/1-59259-364-x:111

2. Ihaka R, Gentleman R. R: A Language for Data Analysis and Graphics. Journal of Computational and Graphical Statistics. 1996;5: 299–314.

3. Yang YH, Dudoit S, Luu P, Lin DM, Peng V, et al. Normalization for cDNA microarray data: a robust composite method addressing single and multiple slide systematic variation. Nucleic Acids Res. 2002;30: e15–e15. doi:10.1093/nar/30.4.e15

4. Lönnstedt I, Speed T. Replicated Microarray Data. Statistica Sinica. 2002;12: 31–46.

5. Snel B, Lehmann G, Bork P, Huynen M. STRING: a web-server to retrieve and display the repeatedly occurring neighbourhood of a gene. Nucleic Acids Res. 2000;28: 3442–3444. doi:10.1093/nar/28.18.3442

6. Zhou Y, Zhou B, Pache L, Chang M, Khodabakhshi AH, et al. Metascape provides a biologist-oriented resource for the analysis of systems-level datasets. Nat Commun. 2019;10: 1523. doi:10.1038/s41467-019-09234-6
