# Supplementary figures and images for: An Arginine-Rich Motif in the ORF2 capsid protein regulates the hepatitis E virus lifecycle and interactions with the host cell
Source: PLoS Pathog. 2022 Aug 25;18(8):e1010798. doi: 10.1371/journal.ppat.1010798 (PMC9451086; doi:10.1371/journal.ppat.1010798)

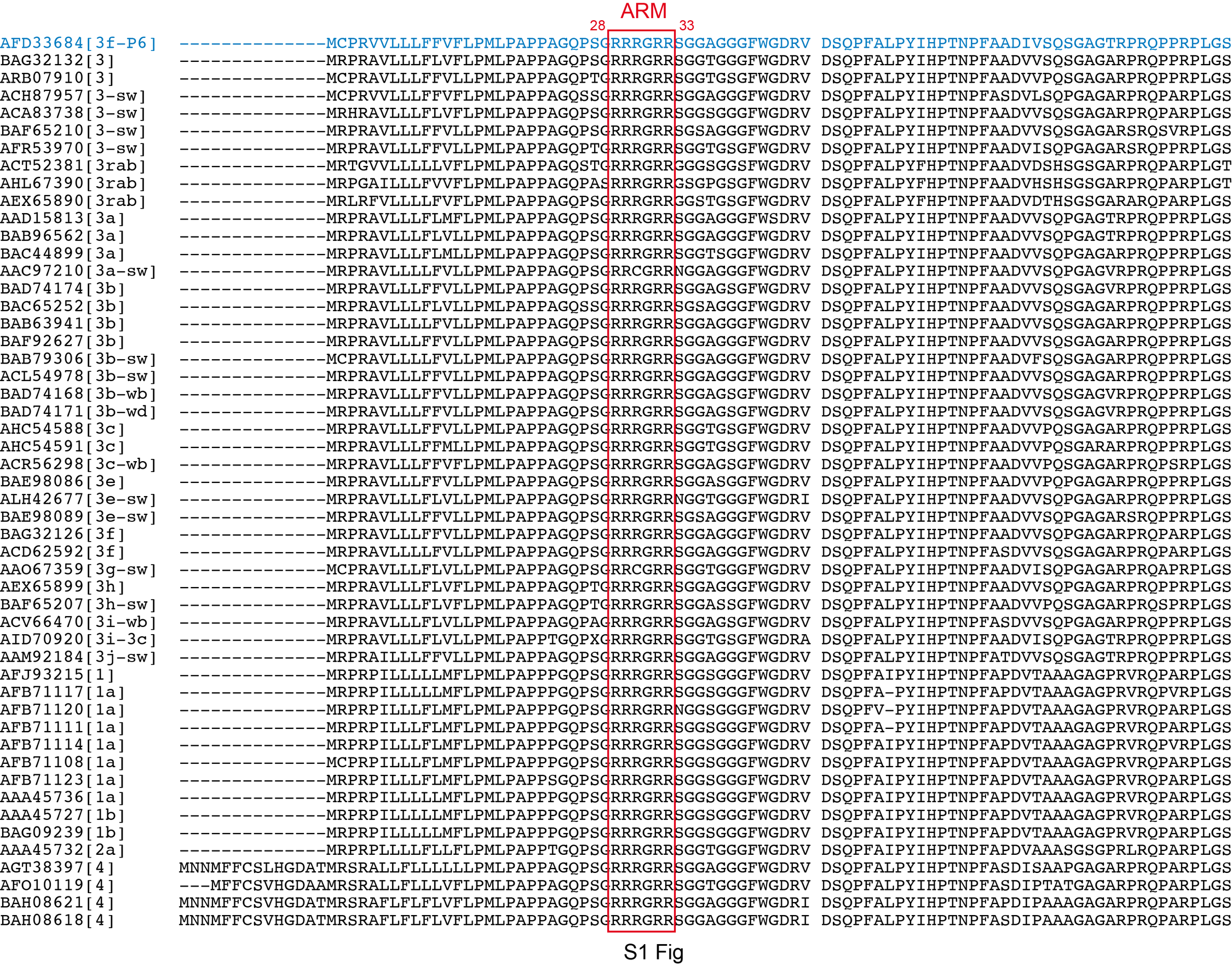

Supplement: S1 Fig — (TIF) [file ppat.1010798.s002.tif]

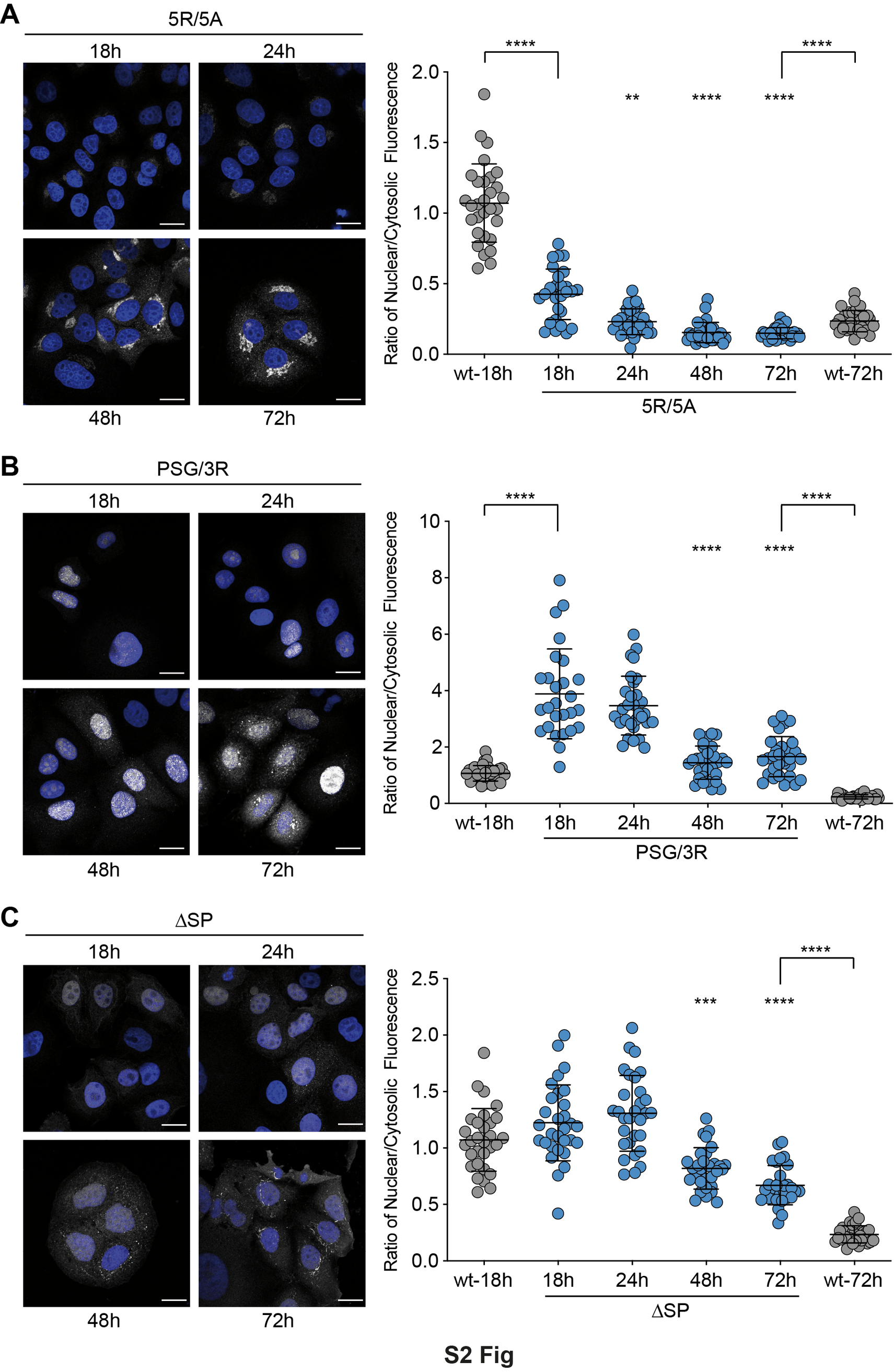

Supplement: S2 Fig — (TIF) [file ppat.1010798.s003.tif]

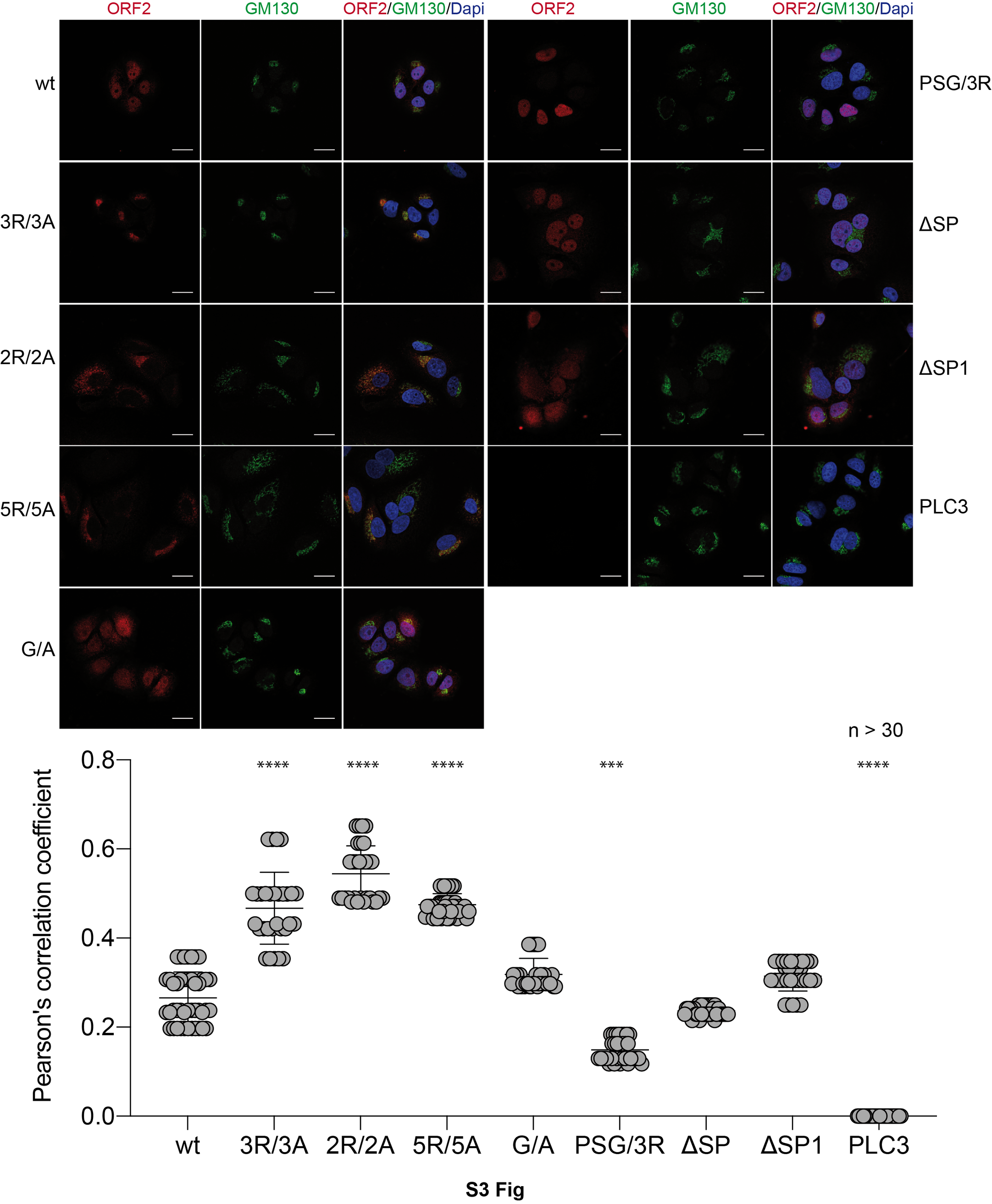

Supplement: S3 Fig — (TIF) [file ppat.1010798.s004.tif]

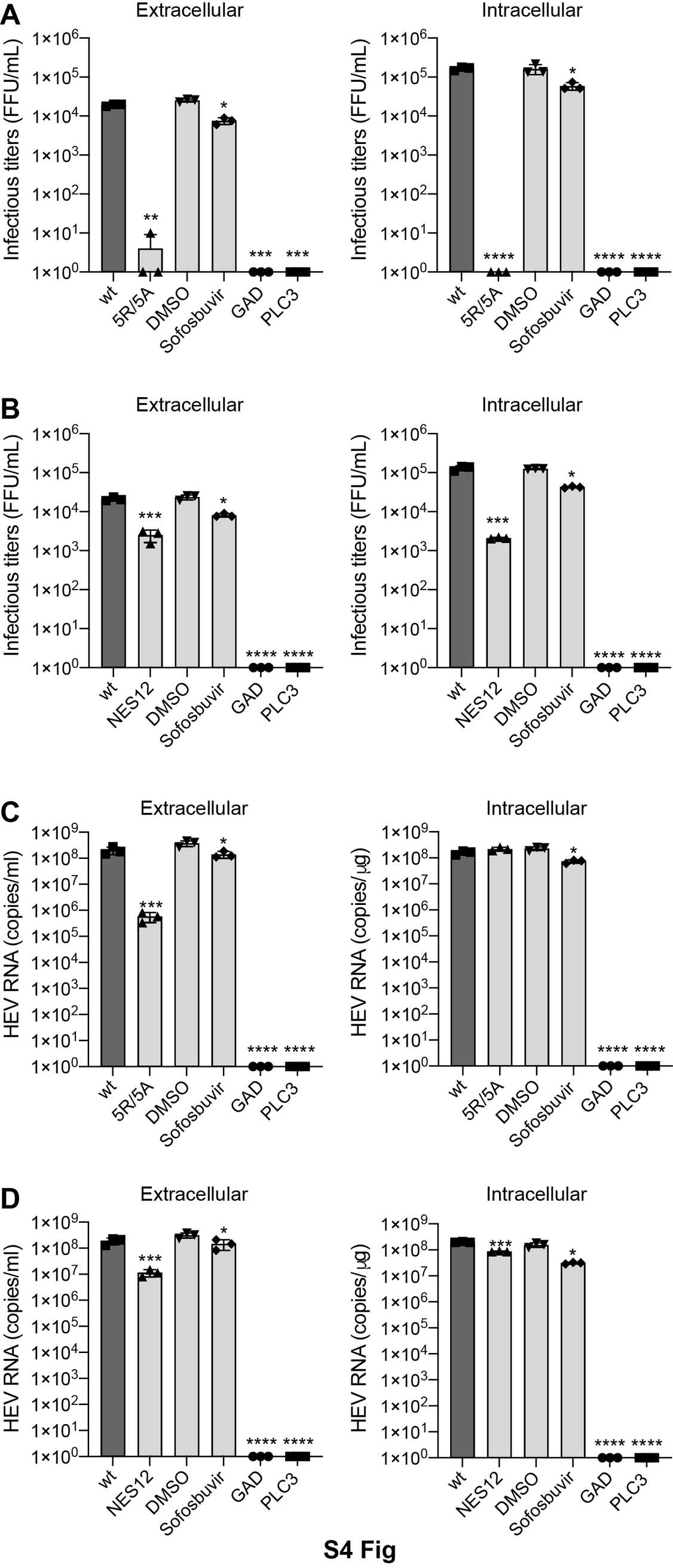

Supplement: S4 Fig — (TIF) [file ppat.1010798.s005.tif]

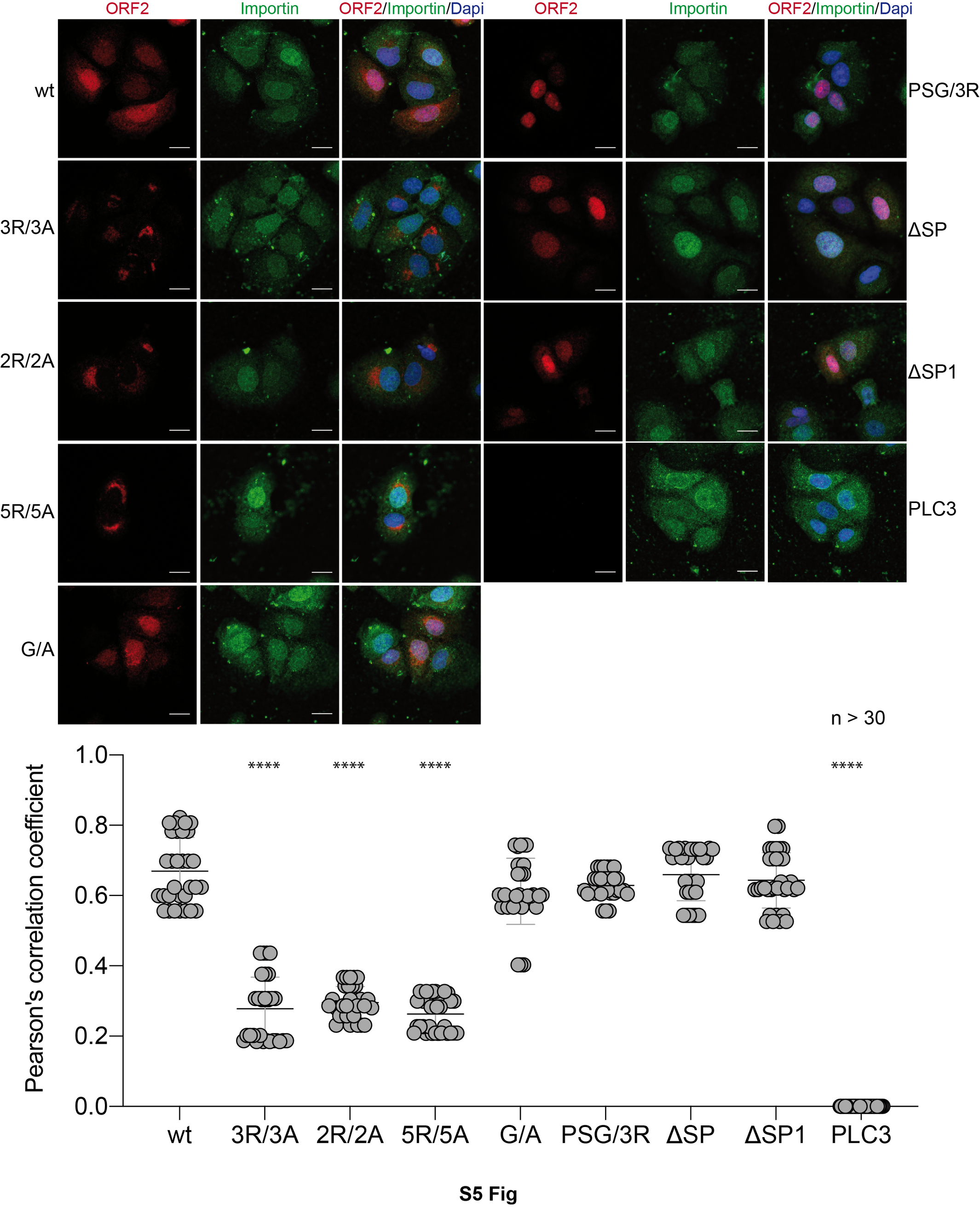

Supplement: S5 Fig — (TIF) [file ppat.1010798.s006.tif]

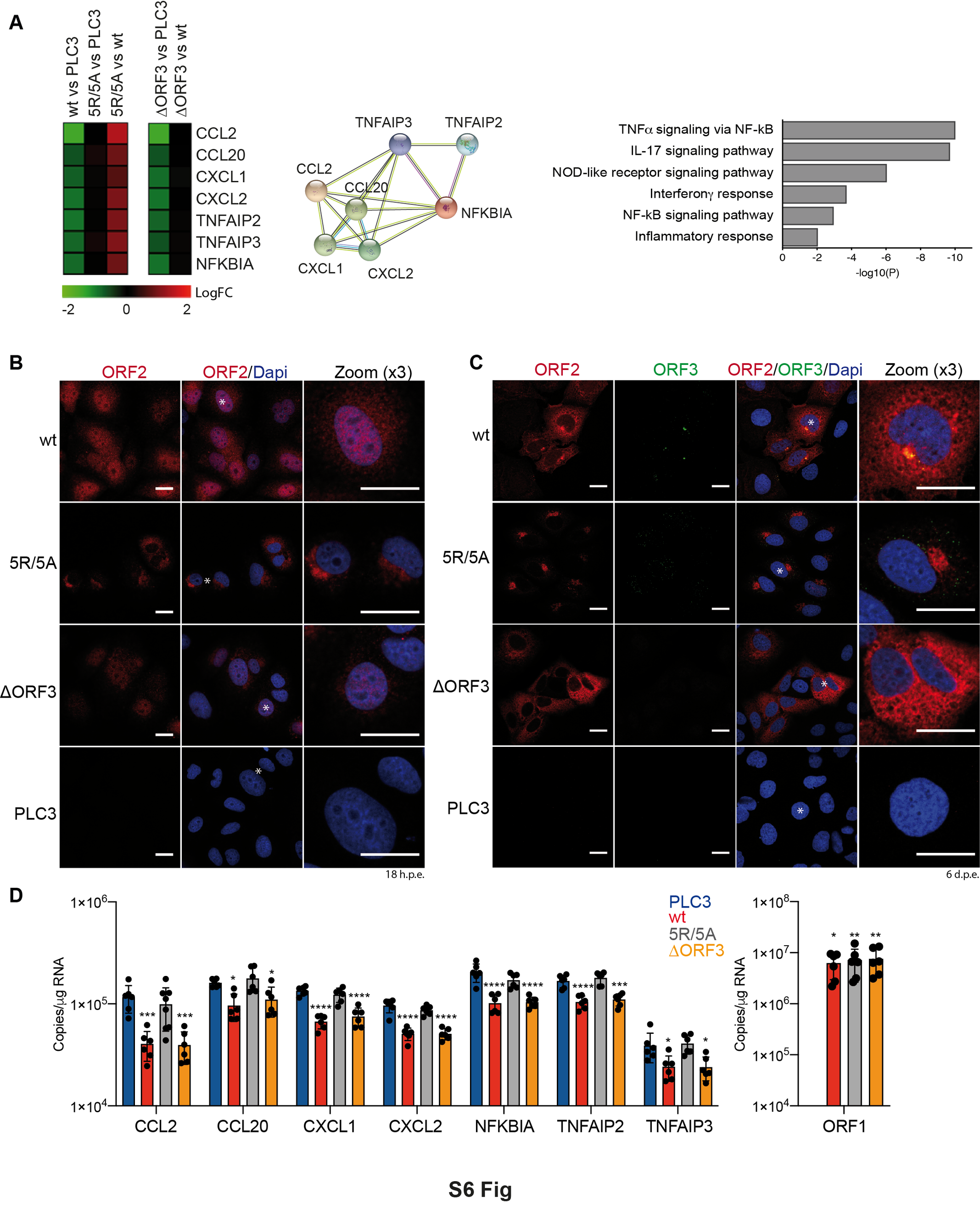

Supplement: S6 Fig — (TIF) [file ppat.1010798.s007.tif]

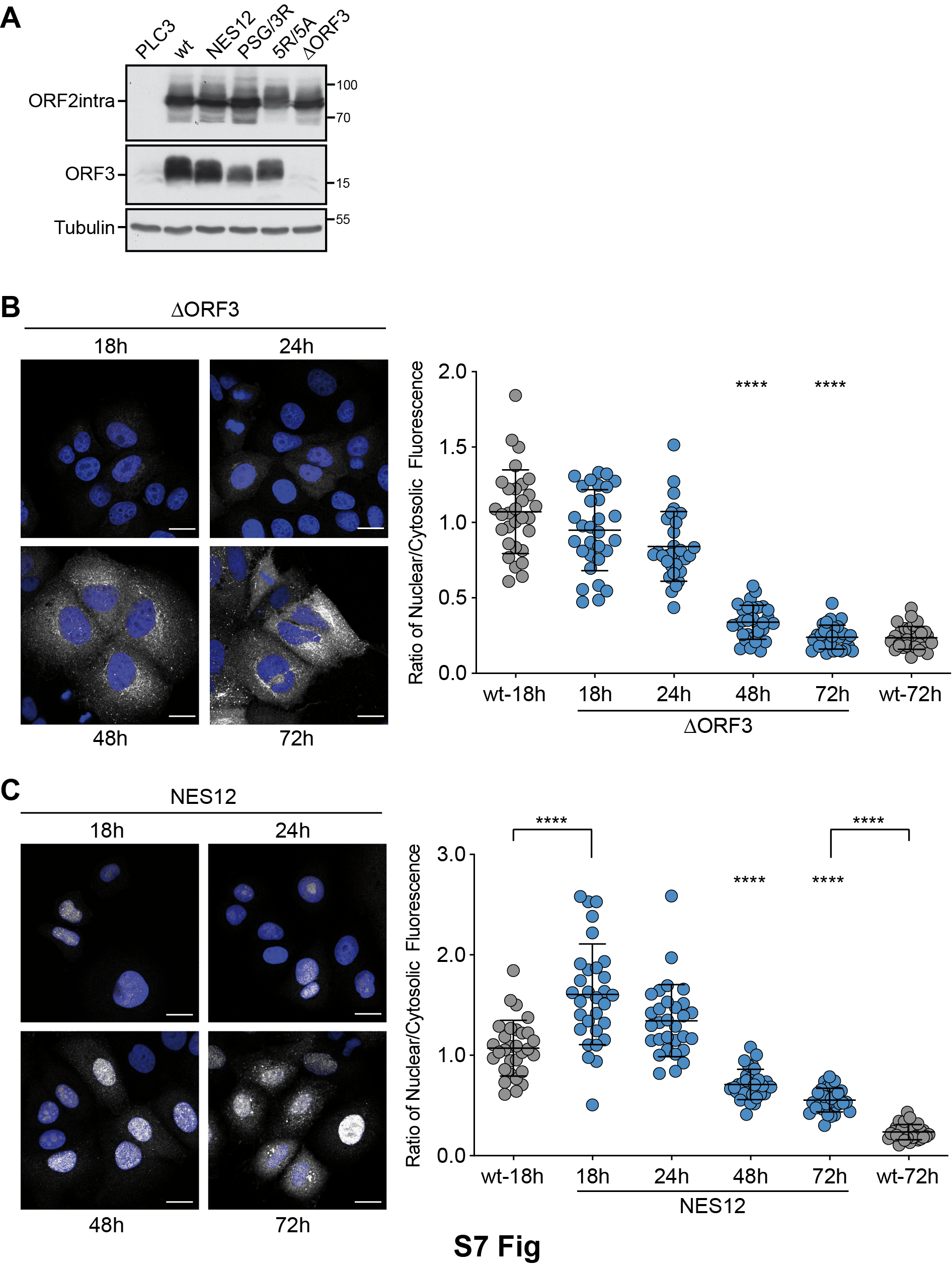

Supplement: S7 Fig — (TIF) [file ppat.1010798.s008.tif]

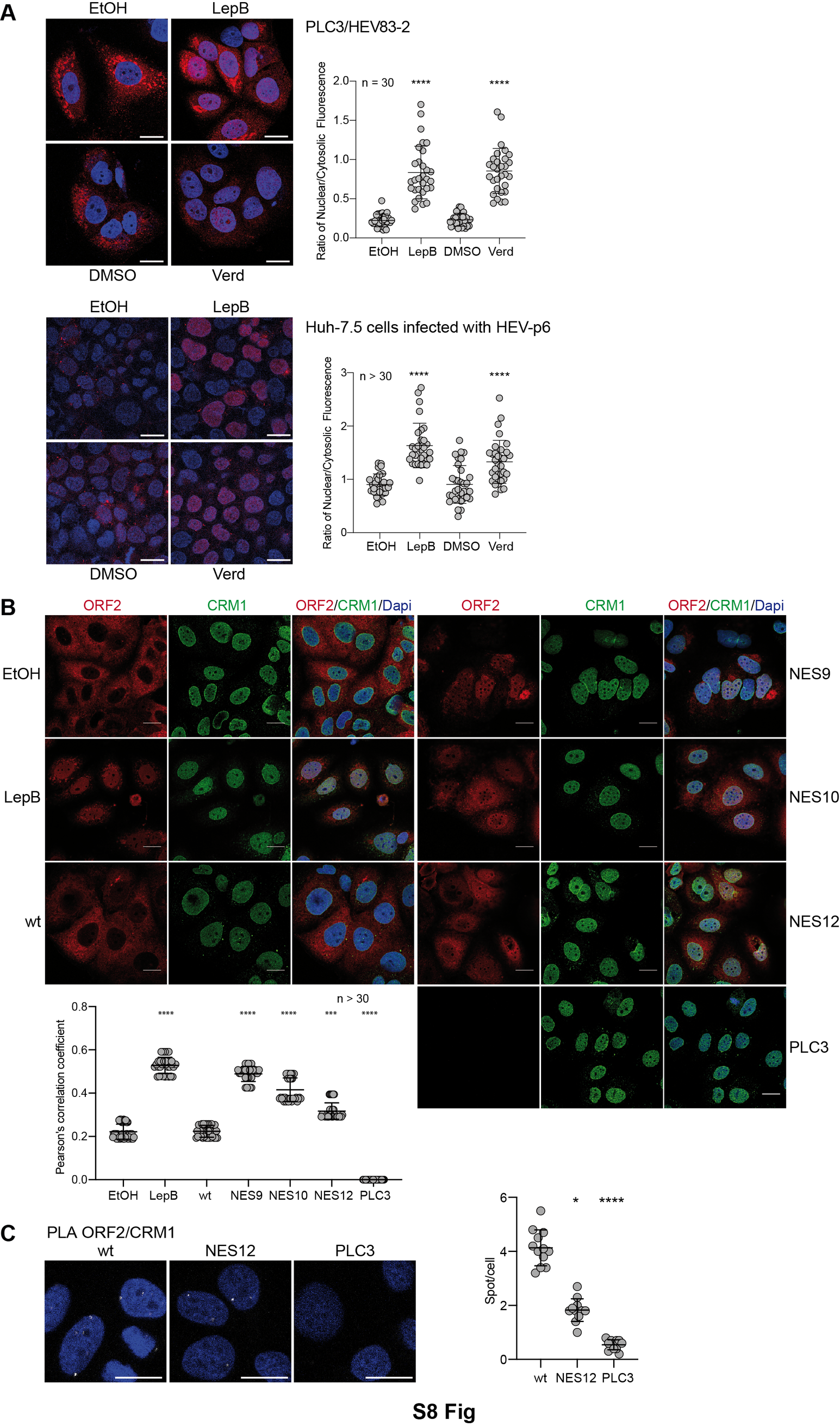

Supplement: S8 Fig — (TIF) [file ppat.1010798.s009.tif]

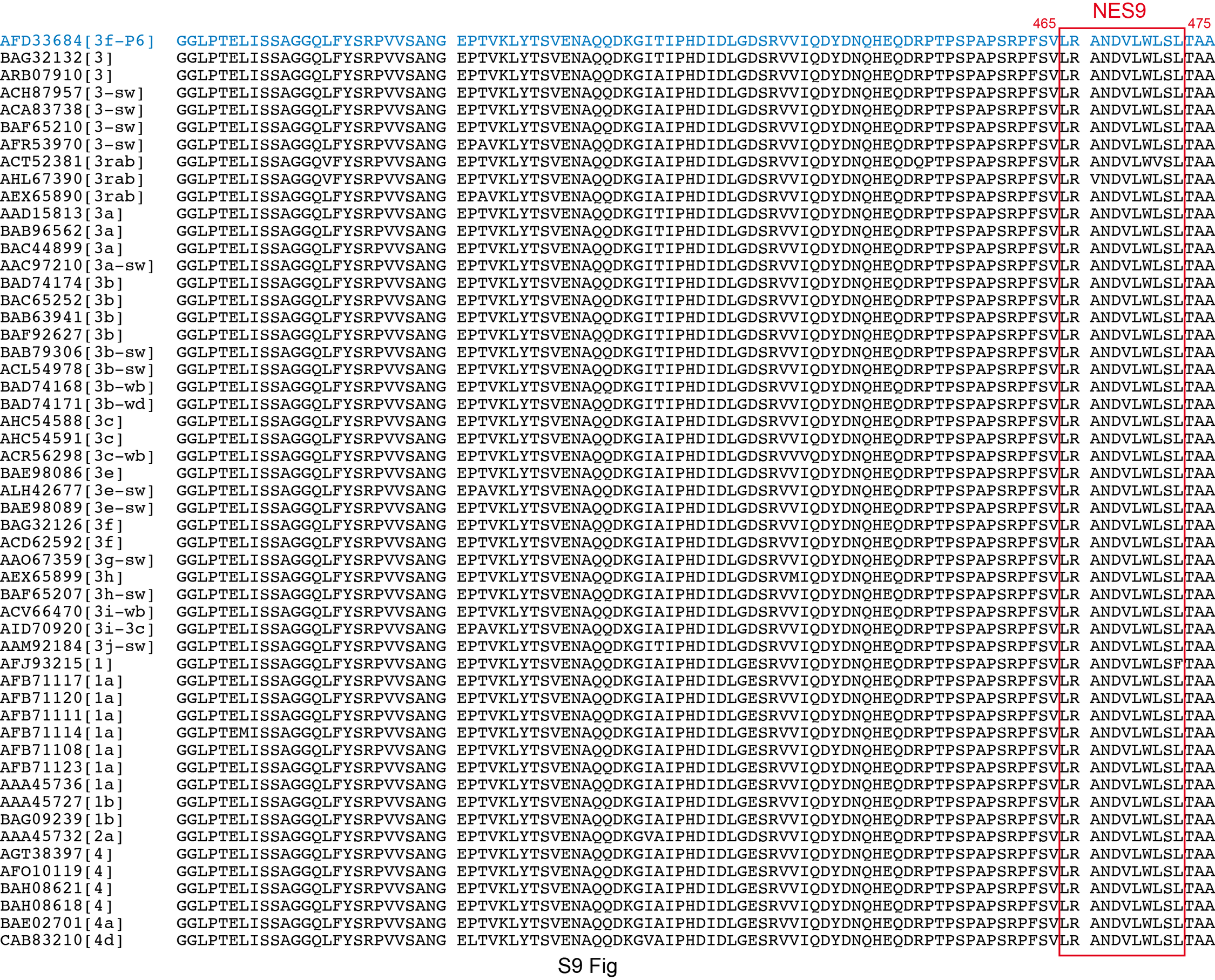

Supplement: S9 Fig — (TIF) [file ppat.1010798.s010.tif]

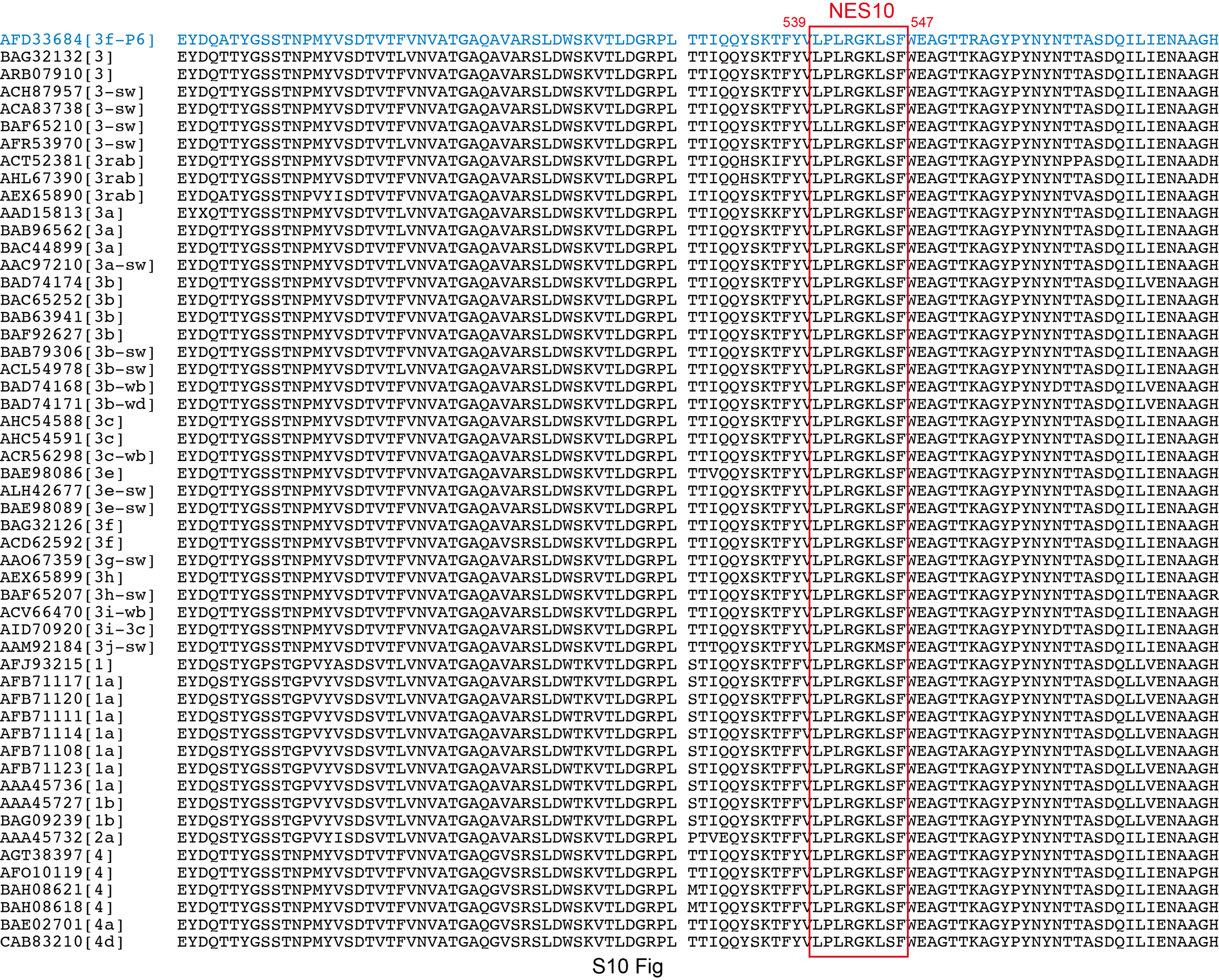

Supplement: S10 Fig — (TIF) [file ppat.1010798.s011.tif]

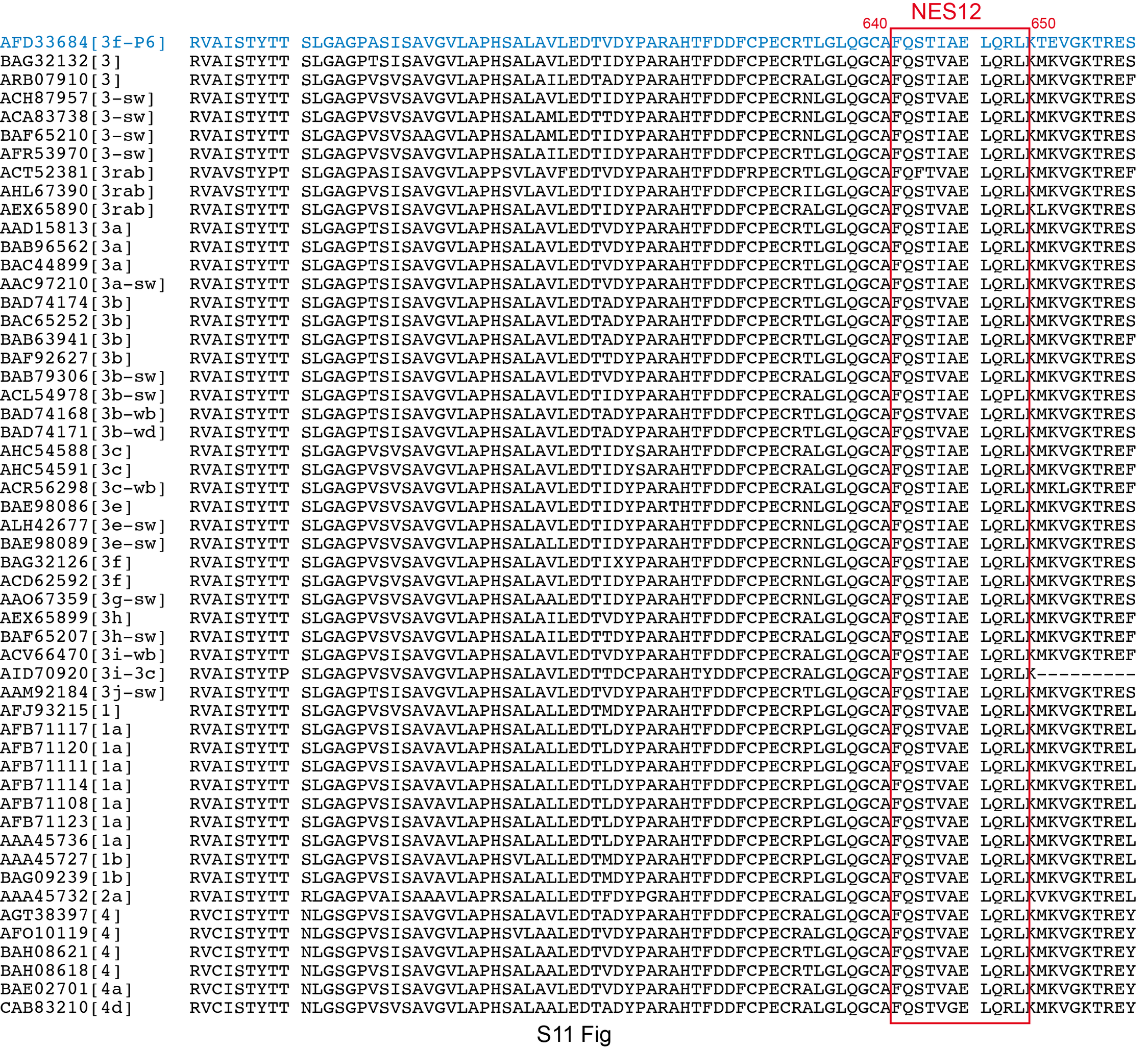

Supplement: S11 Fig — (TIF) [file ppat.1010798.s012.tif]

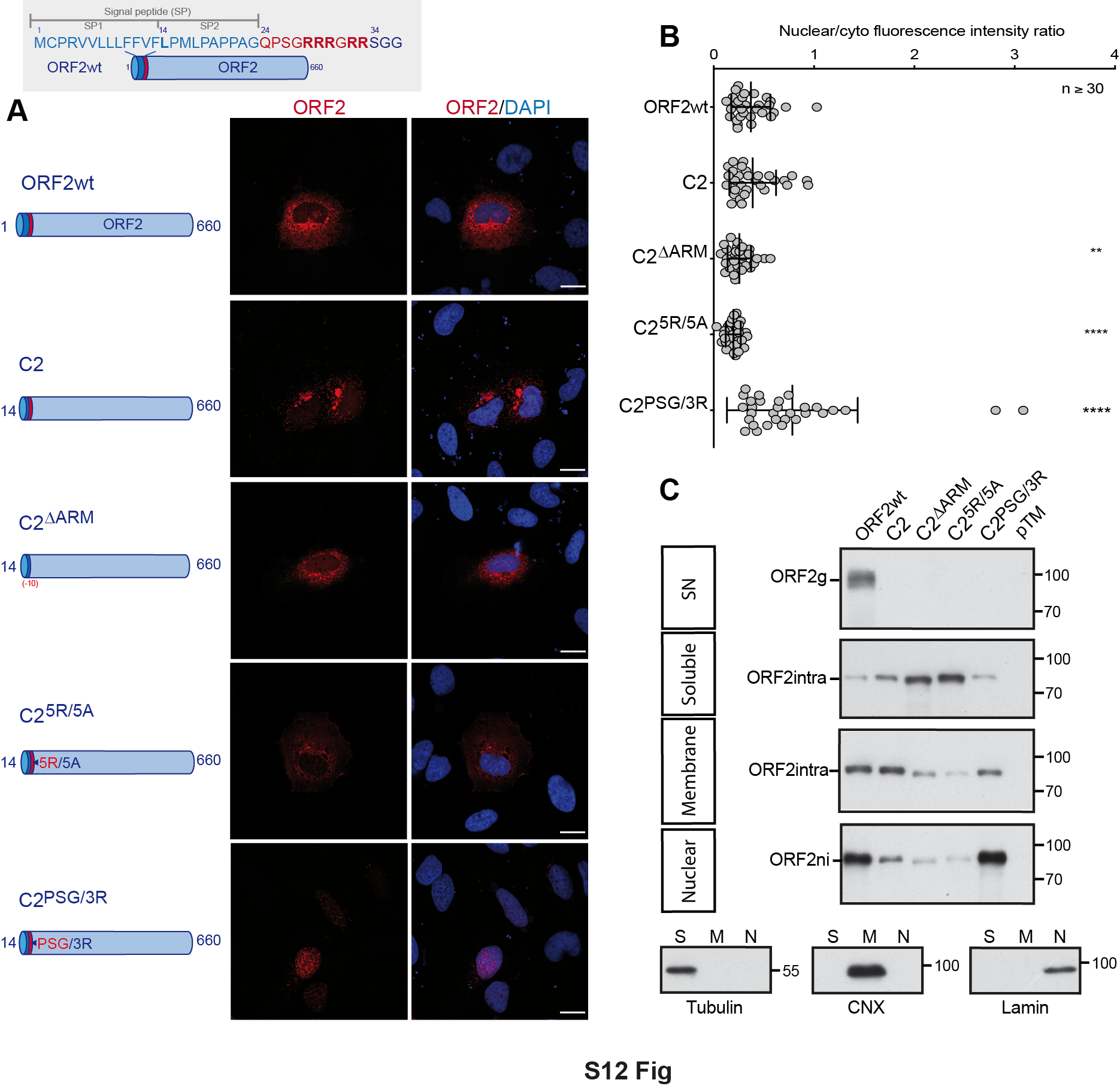

Supplement: S12 Fig — (TIF) [file ppat.1010798.s013.tif]

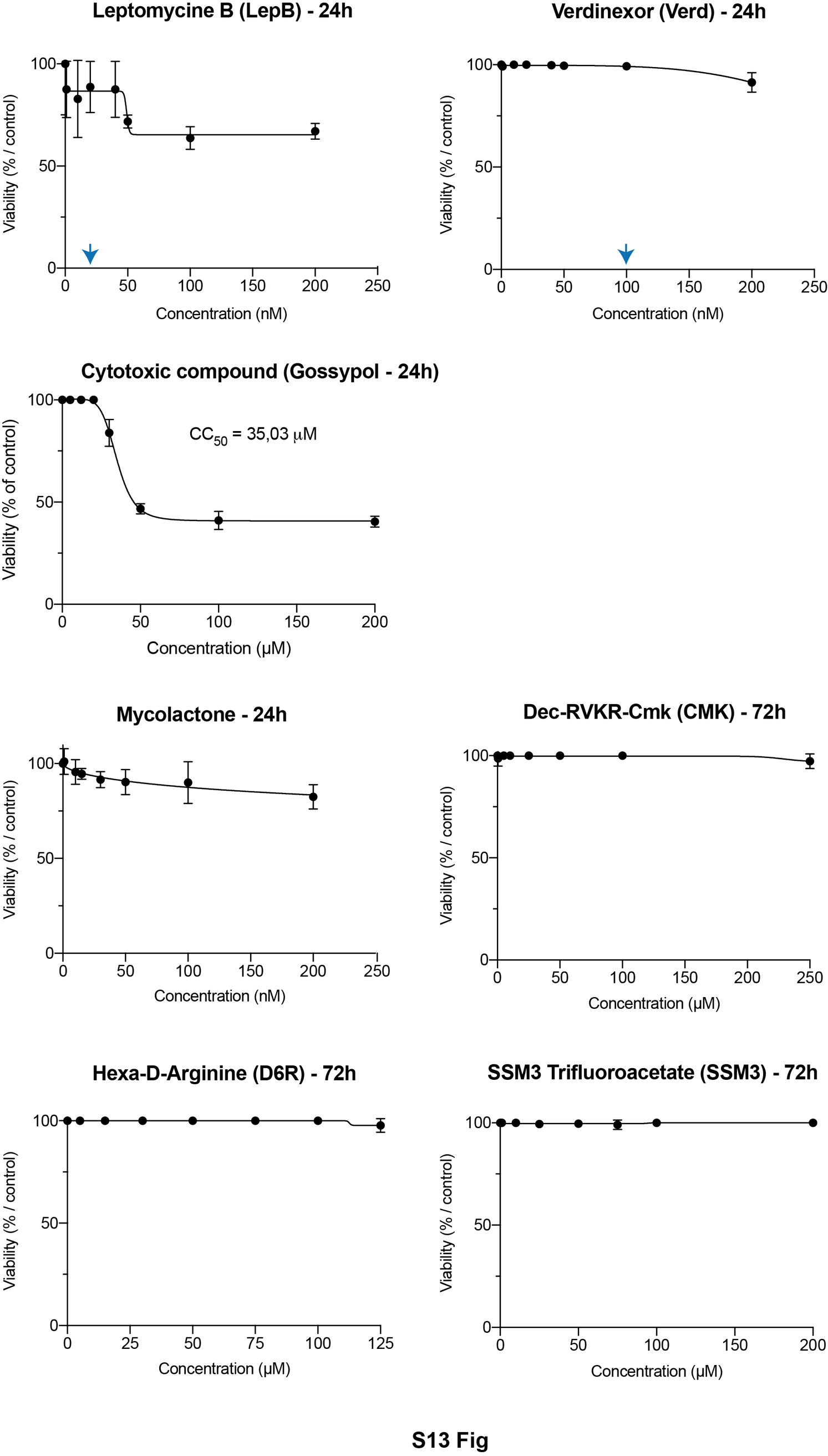

Supplement: S13 Fig — (TIF) [file ppat.1010798.s014.tif]

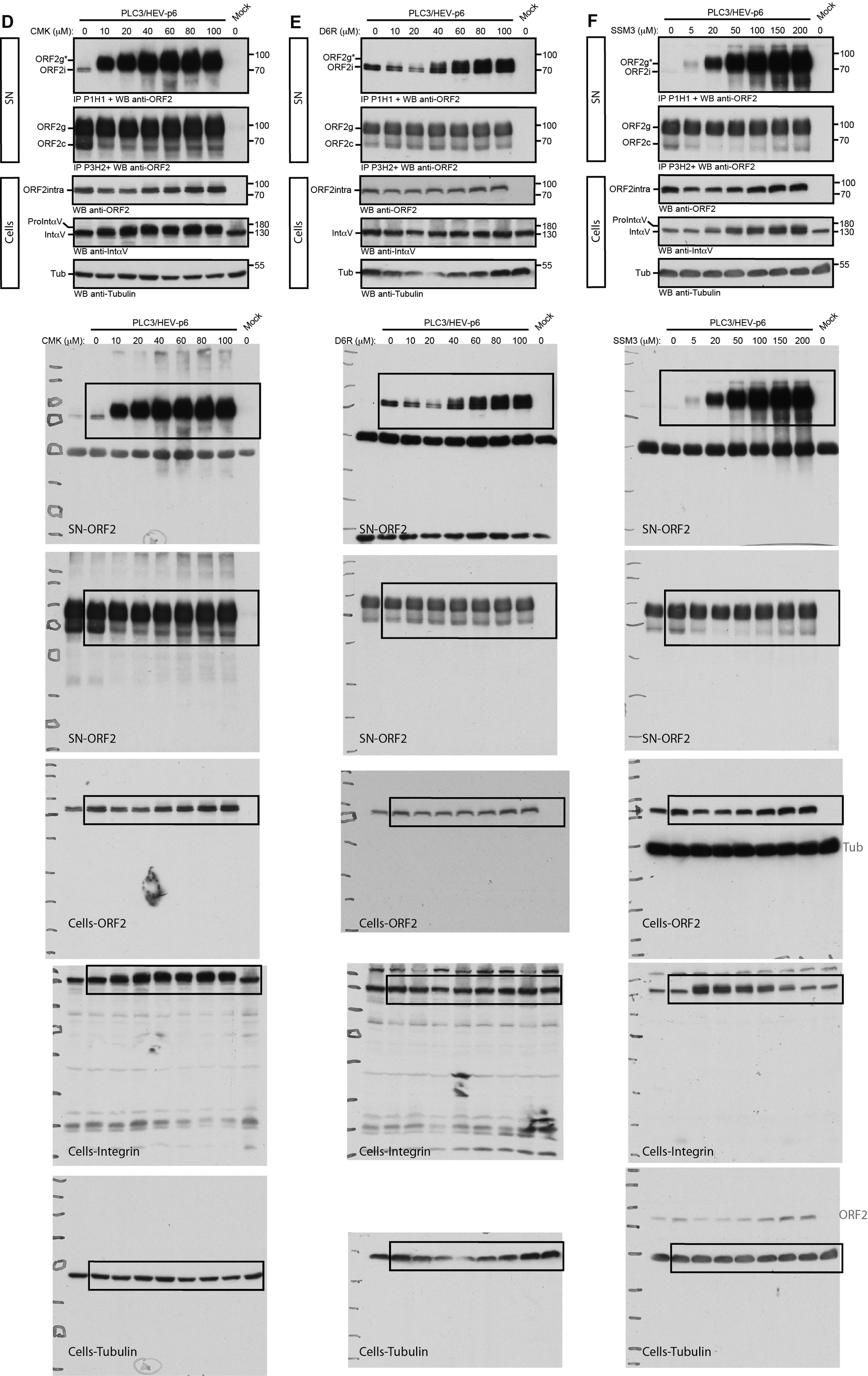

Supplement: S2 Data — (ZIP) [file ppat.1010798.s018.zip › Data S2-Uncropped gels/Uncropped Gels - FIG4 D-F.tif]

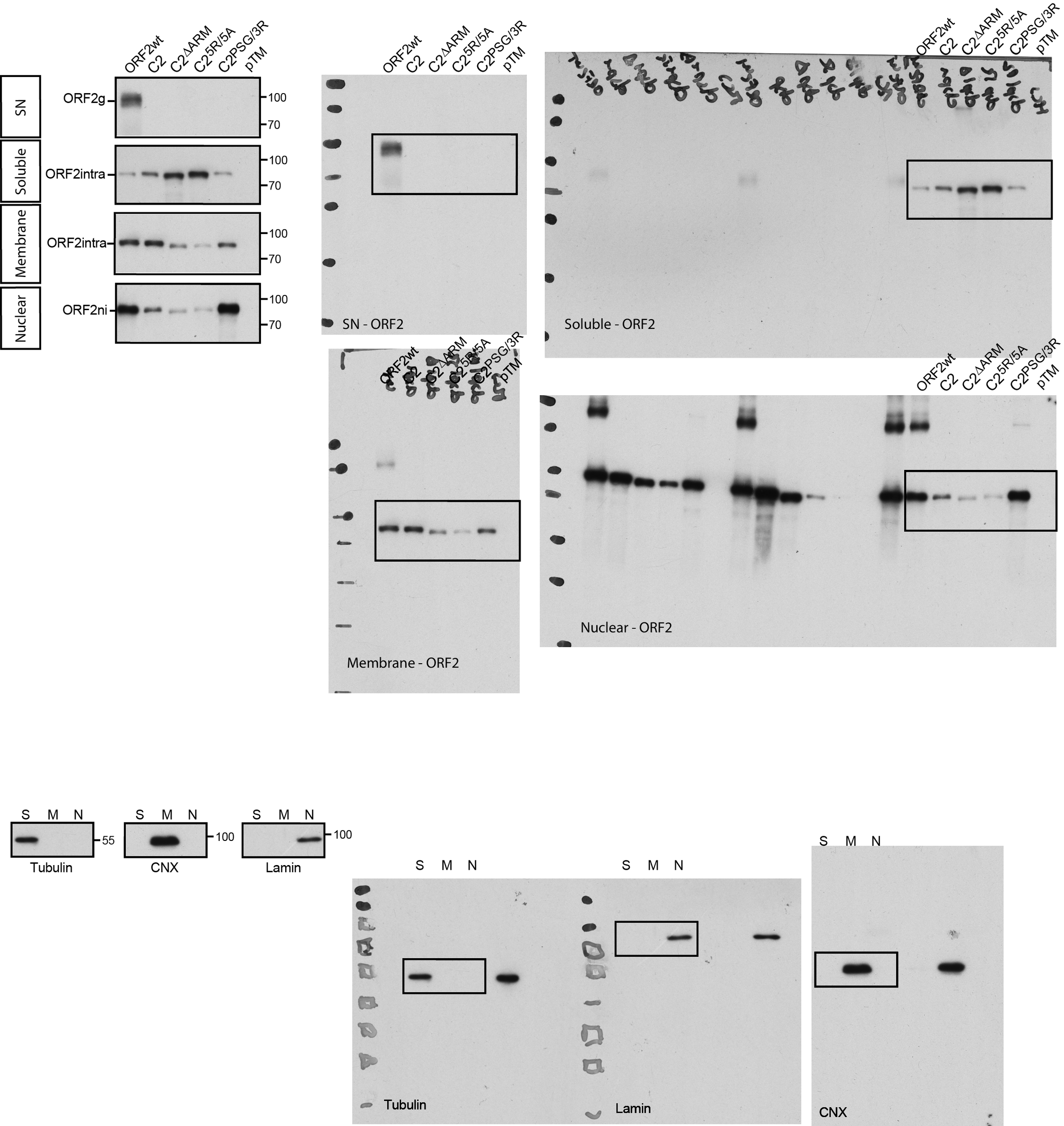

Supplement: S2 Data — (ZIP) [file ppat.1010798.s018.zip › Data S2-Uncropped gels/Uncropped Gels - S12 FIG .tif]

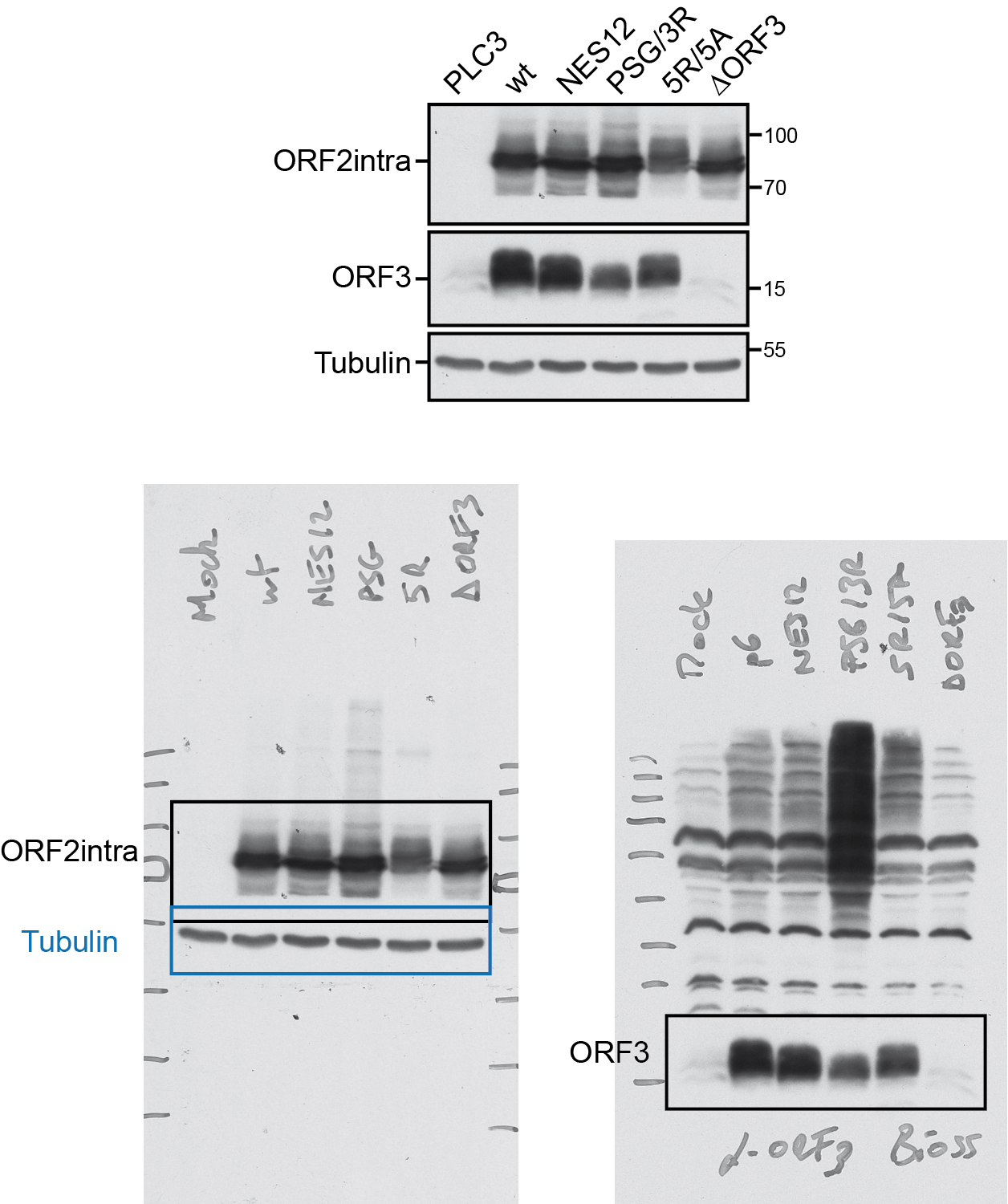

Supplement: S2 Data — (ZIP) [file ppat.1010798.s018.zip › Data S2-Uncropped gels/Uncropped Gels - S7A FIG.tif]

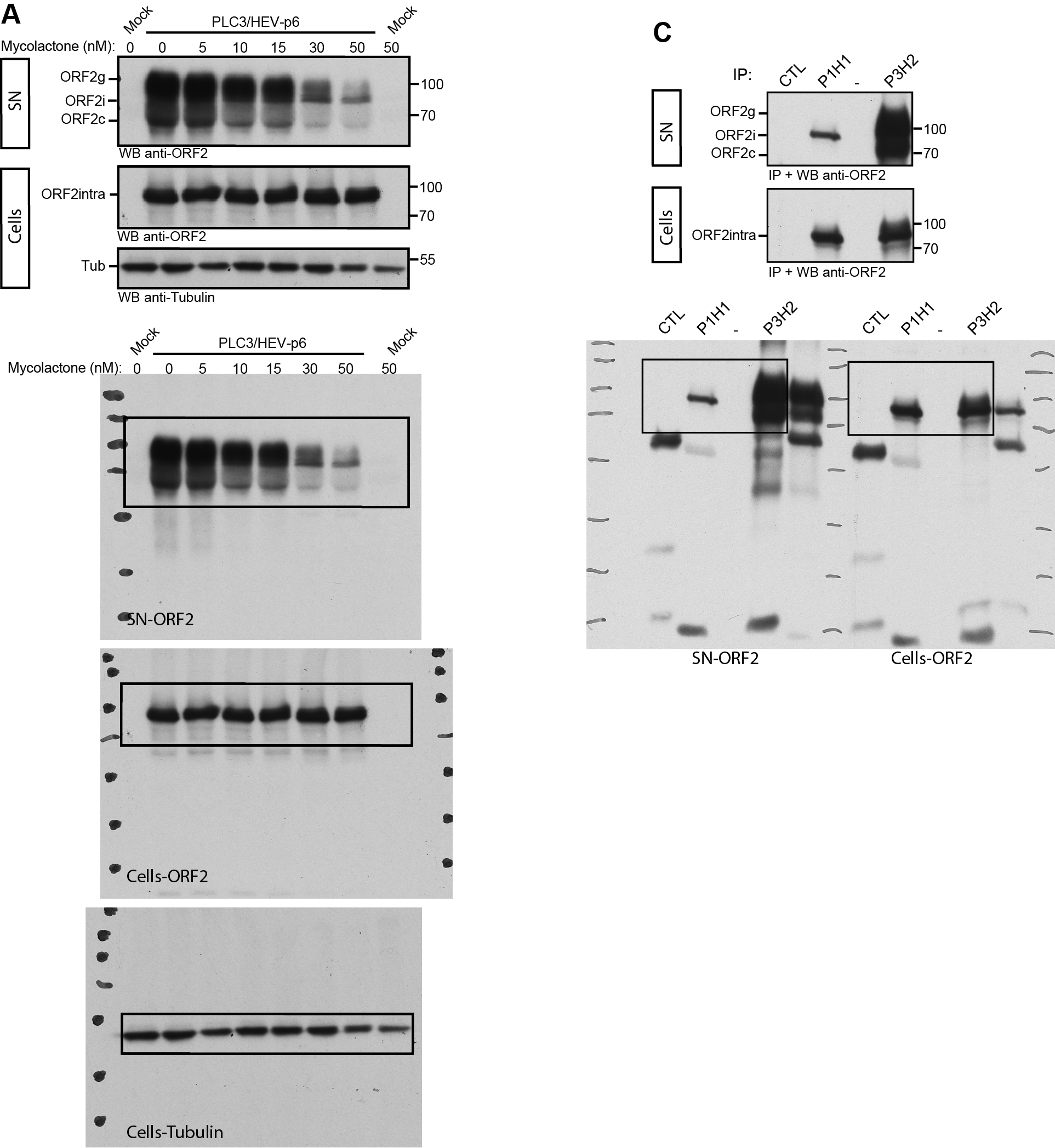

Supplement: S2 Data — (ZIP) [file ppat.1010798.s018.zip › Data S2-Uncropped gels/Uncropped Gels - FIG4 A,C.tif]

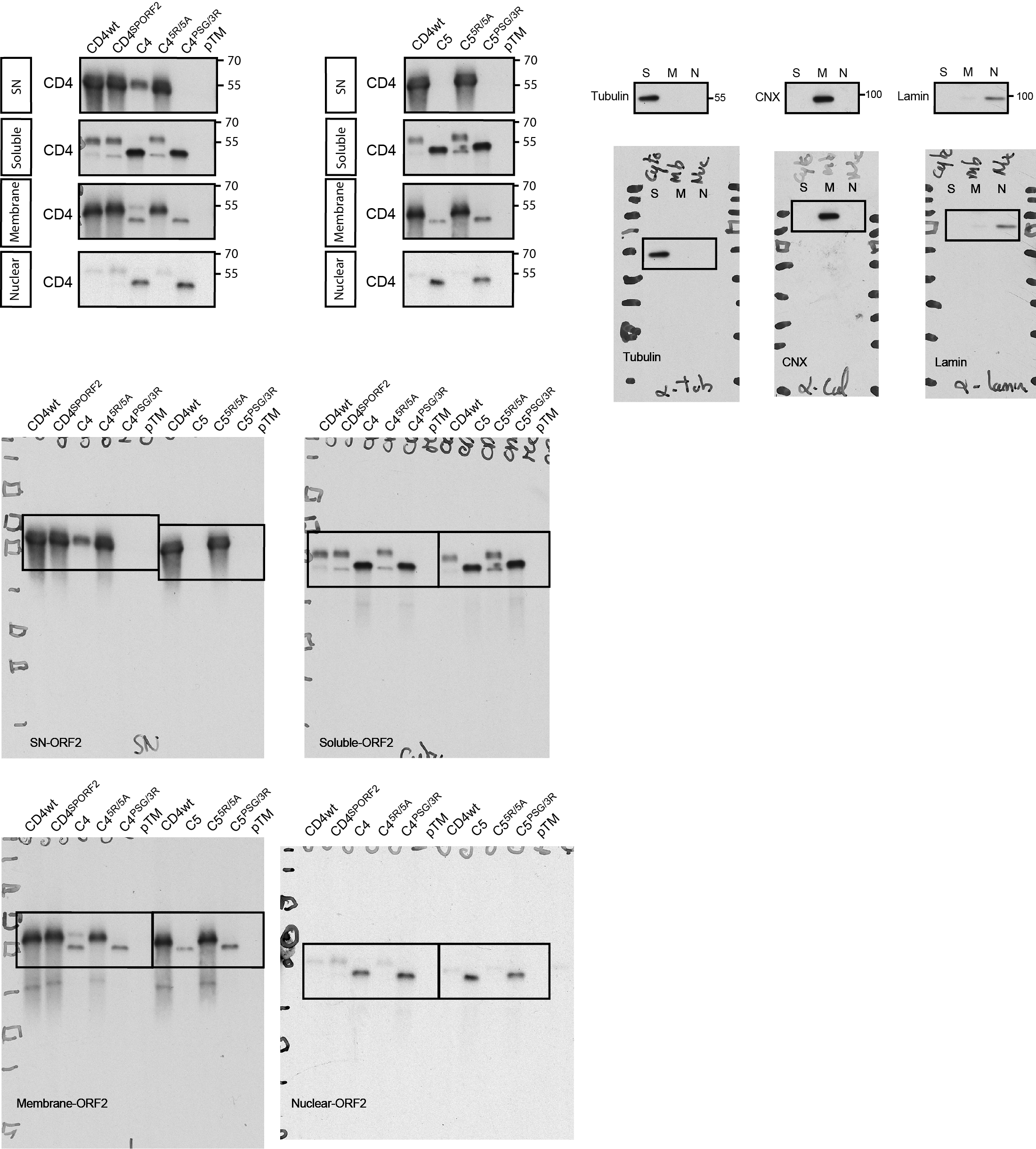

Supplement: S2 Data — (ZIP) [file ppat.1010798.s018.zip › Data S2-Uncropped gels/Uncropped Gels - FIG6D.tif]

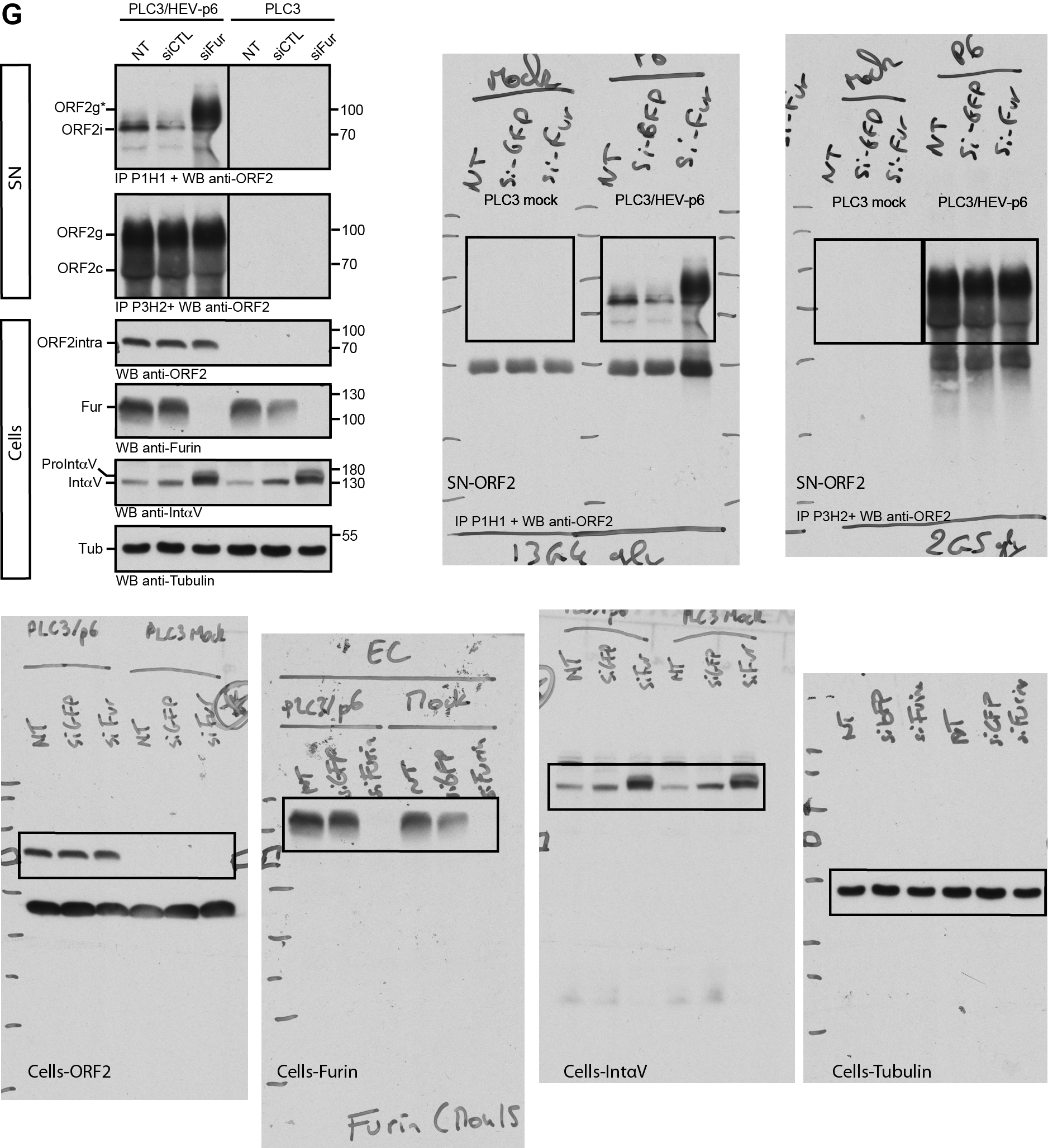

Supplement: S2 Data — (ZIP) [file ppat.1010798.s018.zip › Data S2-Uncropped gels/Uncropped Gels - FIG4 G.tif]

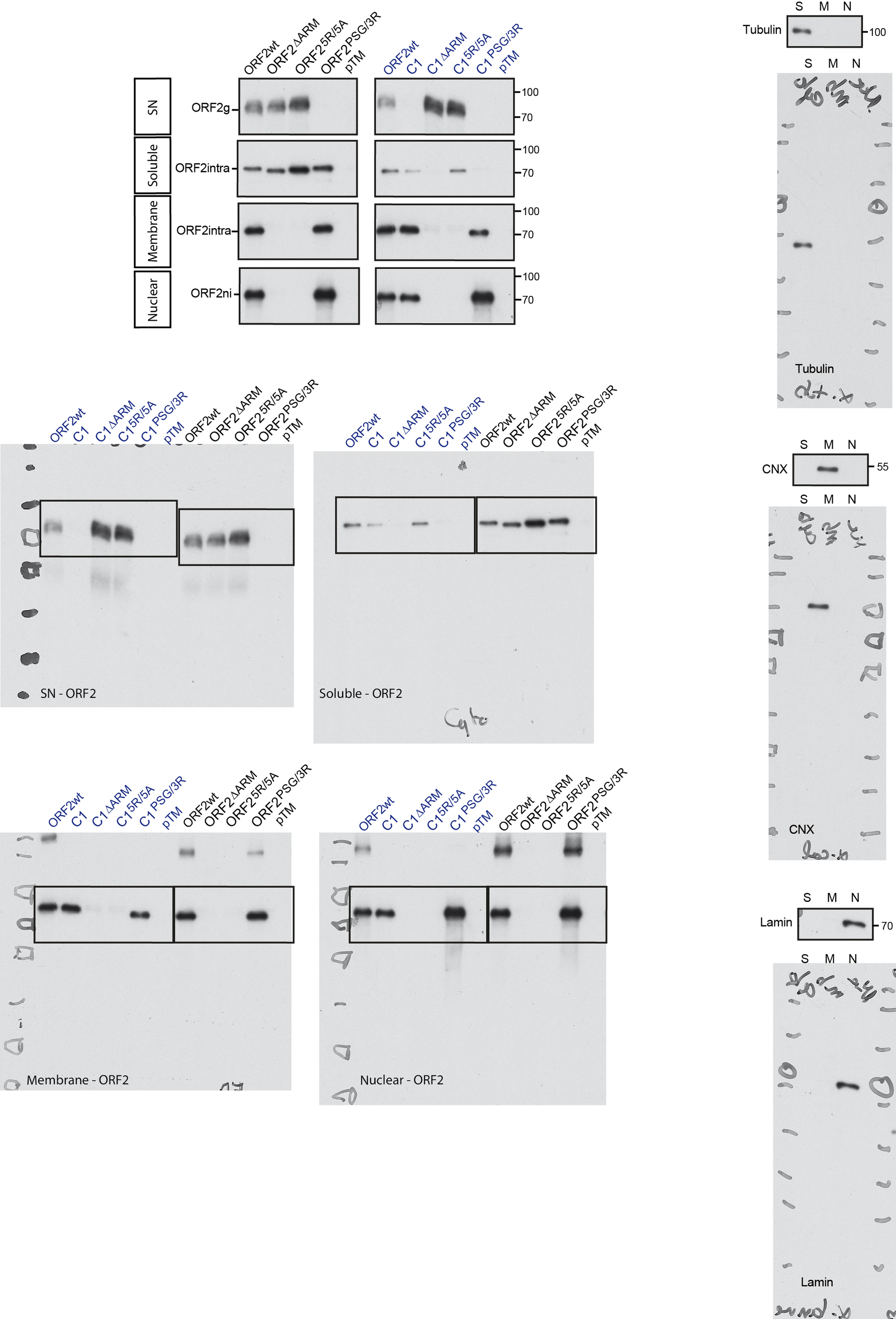

Supplement: S2 Data — (ZIP) [file ppat.1010798.s018.zip › Data S2-Uncropped gels/Uncropped Gels - FIG5C.tif]

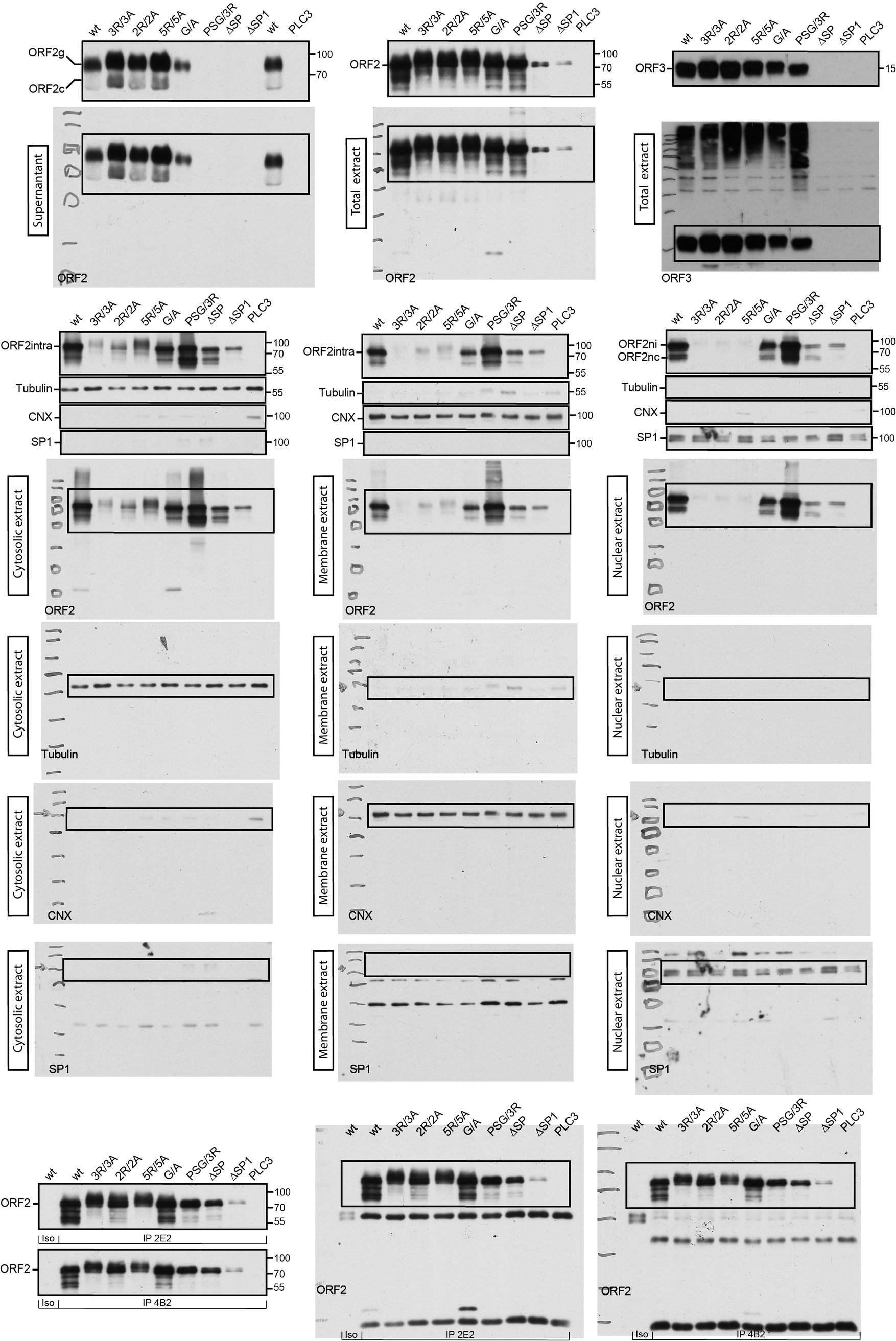

Supplement: S2 Data — (ZIP) [file ppat.1010798.s018.zip › Data S2-Uncropped gels/Uncropped Gels - FIG2C.tif]

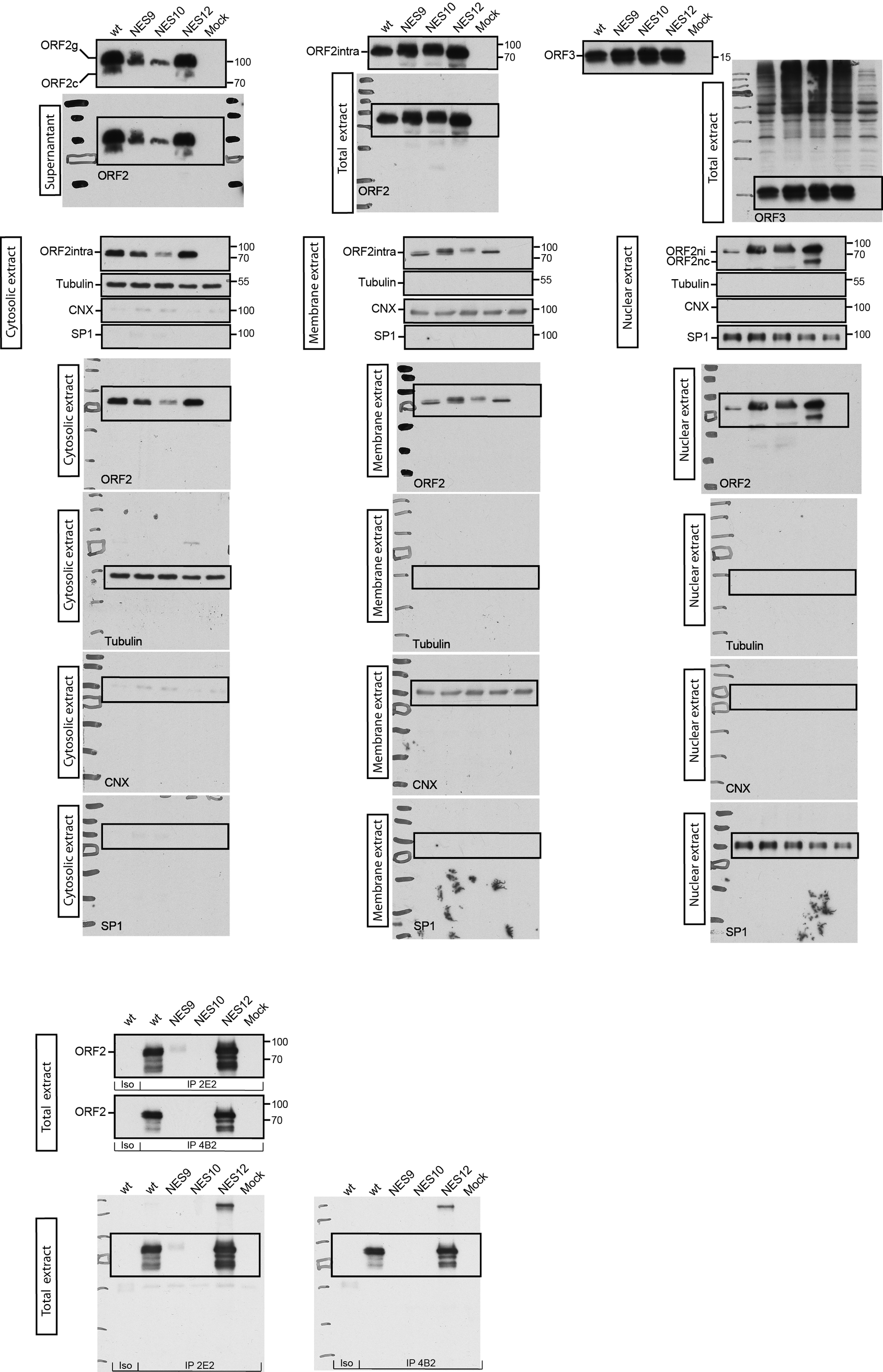

Supplement: S2 Data — (ZIP) [file ppat.1010798.s018.zip › Data S2-Uncropped gels/Uncropped Gels - FIG3D.tif]
