# Supplementary material for: An Arginine-Rich Motif in the ORF2 capsid protein regulates the hepatitis E virus lifecycle and interactions with the host cell
Source: PLoS Pathog. 2022 Aug 25;18(8):e1010798. doi: 10.1371/journal.ppat.1010798 (PMC9451086; doi:10.1371/journal.ppat.1010798)
Supplement: S1 Table — (DOCX) [file ppat.1010798.s015.docx]

**S1 Table: Primers of use to generate ORF2/CD4 chimeras/mutants.**

| **Name**  **(Orientation)** | **Sequence** | **Corresponding mutant/group** |
| --- | --- | --- |
| HEV-1 (Fw) | TTTCTGCCTATGCTGCCCGCGCCACCGGCCGGCCAGCCGTCTGGCGCTGCTGCTGGGCGGCGCAGCGGCGGTGCCGGCGGTGGTTTCTGGGGTGACAGG | 3R/3A |
| HEV-2 (Rev) | CCTGTCACCCCAGAAACCACCGCCGGCACCGCCGCTGCGCCGCCCAGCAGCAGCGCCAGACGGCTGGCCGGCCGGTGGCGCGGGCAGCATAGGCAGAAA |  |
| HEV-3 (Fw) | CGTCGTCGTGGGGCGGCCAGCGGCGGTGC | 2R/2A |
| HEV-4 (Rev) | GCACCGCCGCTGGCCGCCCCACGACGACG |  |
| HEV-5 (Fw) | CCGTCTGGCGCTGCTGCTGGGGCGGCCAGCGGCGGTGCCGGCGGT | 5R/5A |
| HEV-6 (Rev) | ACCGCCGGCACCGCCGCTGGCCGCCCCAGCAGCAGCGCCAGACGG |  |
| HEV-7 (Fw) | TCTGGCCGTCGTCGTGCGCGGCGCAGCGGCGGT | G/A |
| HEV-8 (Rev) | ACCGCCGCTGCGCCGCGCACGACGACGGCCAGA |  |
| HEV-9 (Fw) | CCCGCGCCACCGGCCGGCCAGCGGCGTCGCCGTCGTCGTGGGCGGCGC | PSG/3R |
| HEV-10 (Rev) | GCGCCGCCCACGACGACGGCGACGCCGCTGGCCGGCCGGTGGCGCGGG |  |
| HEV-11 (Fw) | CAGCCGTCTGGCCGTCGTCG | ΔSP |
| HEV-12 (Rev) | ACGGCCAGACGGCTGCATGGTGATCCCATGGGCGATGCAACA |  |
| HEV-13 (Fw) | CTGCCTATGCTGCCCGCGCCACCGGCCGGCCAG | ΔSP1 |
| HEV-14 (Rev) | GGGCAGCATAGGCAGCATGGTGATCCCATGGGCGATGCAACAAACATGTTATTCATT |  |
| HEV-15 (Fw) | CCTGCCCCCTCACGCCCTTTCTCAGTCGCTCGCGCTAACGATGCTTTGTGGGCCTCCGCCACTGCCGCTGAGTACGATCAGGCTACG | NES9 |
| HEV-16 (Rev) | CGTAGCCTGATCGTACTCAGCGGCAGTGGCGGAGGCCCACAAAGCATCGTTAGCGCGAGCGACTGAGAAAGGGCGTGAGGGGGCAGG |  |
| HEV-17 (Fw) | CAGCAGTATTCTAAGACATTTTATGTTGCCCCGGCCCGCGGGAAGGCGTCCGCTTGGGAGGCTGGCACAACTAGGGCCGGC | NES10 |
| HEV-18 (Rev) | GCCGGCCCTAGTTGTGCCAGCCTCCCAAGCGGACGCCTTCCCGCGGGCCGGGGCAACATAAAATGTCTTAGAATACTGCTG |  |
| HEV-19 (Fw) | CGTACCCTAGGTTTGCAGGGTTGTGCAGCCCAGTCCACTGCTGCTGAGGCTCAGCGCGCTAAAACGGAGGTAGGCAAAACCCGGGAG | NES12 |
| HEV-20 (Rev) | CTCCCGGGTTTTGCCTACCTCCGTTTTAGCGCGCTGAGCCTCAGCAGCAGTGGACTGGGCTGCACAACCCTGCAAACCTAGGGTACG |  |
| HEV-21 (Fw) | GATATGTACAACCATGCTGCCTATGCTGCCCGCGCC | ORF2wt |
| HEV-22 (Fw) | GATATGTACAACCATGTGCCCTAGGGTTGTTCTGCTGCTGTTCTTCGTGTTTCTGCCTATGCTGCCCGCGCCACCGGCCGGCAGCGGCGGTGCCGGCGGTGGTTTCTGGGGTG |  |
| HEV-23 (Fw) | GATA TGTACA ACC ATGTGCCCTAGGGTTGTTCTGC | C2 |
| HEV-24 (Fw) | GATATGTACAACCATGCTGCCTATGCTGCCCGCGCCACCGGCCGGCAGCGGCGGTGCCGGCGGTGGTTTCTGGGG |  |
| HEV-29(Fw) | GATATGTACAACCATGAACCGGGGAGTCCCTTTTAGGCACTTGCTTCTGGTGCTGCAACTGGCGCTCCTCCCAGCAGCCACTCAGGGA | C1 |
| HEV-30 (Fw) | GATATGTACAACCATGAACCGGGGAGTCCCTTTTAGGCACTTGCTTCTGGTGCTGCAACTGGCGCTCCTCCCAGCAGCCACTCAGGGACAGCCGTCTGGCCGTCGTCGTGGGCGGCGC |  |
| HEV-31 (Fw) | GATATGTACAACCATGAACCGGGGAGTCCCTTTTAGGCACTTGCTTCTGGTGCTGCAACTGGCGCTCCTCCCAGCAGCCACTCAGGGACAGCCGTCTGGCGCTGCTGCTGGGGCGGCC |  |
| HEV-32 (Fw) | GATATGTACAACCATGAACCGGGGAGTCCCTTTTAGGCACTTGCTTCTGGTGCTGCAACTGGCGCTCCTCCCAGCAGCCACTCAGGGACAGCGGCGTCGCCGTCGTCGTGGGCGGCGC |  |
| HEV-33 (Fw) | GATAGAATTCACCATGTGCCCTAGGGTTGTTCTGCTGCTGTTCTTCGTGTTTCTGCCTATGCTGCCCGCGCCACCGGCCGGCAAGAAAGTGGTGCTGGGCAAAAAAGGGGATACAGTGG | C4 |
| HEV-34 (Fw) | ATAGAATTCACCATGTGCCCTAGGGTTGTTCTGCTGCTGTTCTTCGTGTTTCTGCCTATGCTGCCCGCGCCACCGGCCGGCCAGCCGTCTGGCCGTCGTCGTGGGCGGCGCAAGAAAGTGGTGCTGGGCAAAAAAGGG |  |
| HEV-35 (Fw) | ATAGAATTCACCATGTGCCCTAGGGTTGTTCTGCTGCTGTTCTTCGTGTTTCTGCCTATGCTGCCCGCGCCACCGGCCGGCCAGCCGTCTGGCGCTGCTGCTGGGGCGGCCAAGAAAGTGGTGCTGGGCAAAAAAGGG |  |
| HEV-36 (Fw) | ATAGAATTCACCATGTGCCCTAGGGTTGTTCTGCTGCTGTTCTTCGTGTTTCTGCCTATGCTGCCCGCGCCACCGGCCGGCCAGCGGCGTCGCCGTCGTCGTGGGCGGCGCAAGAAAGTGGTGCTGGGCAAAAAAGG |  |
| HEV-37 (Fw) | GATAGAATTCACCATGAACCGGGGAGTCCCTTTTAGGCACTTGCTTCTGGTGCTGCAACTGGCGCTCCTCCCAGCAGCCACTCAGGGACAGCCGTCTGGCCGTCGTCGTGGGCGGCGC | C5 |
| HEV-38 (Fw) | GATAGAATTCACCATGAACCGGGGAGTCCCTTTTAGGCACTTGCTTCTGGTGCTGCAACTGGCGCTCCTCCCAGCAGCCACTCAGGGACAGCCGTCTGGCGCTGCTGCTGGGGCGGCC |  |
| HEV-39 (Fw) | GATAGAATTCACCATGAACCGGGGAGTCCCTTTTAGGCACTTGCTTCTGGTGCTGCAACTGGCGCTCCTCCCAGCAGCCACTCAGGGACAGCGGCGTCGCCGTCGTCGTGGGCGGCGC |  |
| HEV-42 (Rev) | GATAGGATCCTTAAGACTCCCGGGTTTTGCCTACC | ORF2 groups |
| HEV-43 (Rev) | GATAACGCGTTCATTAATTAGGCCTCTCGAGCTGC | CD4 groups |
| HEV-44 (Fw) | GCTGTTCTACTCTCGCCC | ORF2 sequencing |
| HEV-45 (Fw) | CGCTACCGCCCGCTGGT |  |
| HEV-46 (Rev) | ACCAGCGGGCGGTAGCG |  |
| HEV-47 (Rev) | GCATTCTCCACAGATGT |  |
| HEV-48 (Rev) | ACTGTAAAGGCGAGTGGG | CD4 sequencing |
| HEV-49 (Fw) | TTCTGGGAAATCAGGGC |  |
