# Supplementary material for: An Arginine-Rich Motif in the ORF2 capsid protein regulates the hepatitis E virus lifecycle and interactions with the host cell
Source: PLoS Pathog. 2022 Aug 25;18(8):e1010798. doi: 10.1371/journal.ppat.1010798 (PMC9451086; doi:10.1371/journal.ppat.1010798)
Supplement: S2 Table — (DOCX) [file ppat.1010798.s016.docx]

**S2 Table: Primers used for RT-qPCR of cellular genes.**

| **Primer name / Target** | **Sequence** |
| --- | --- |
| hCCL2Fw / CCL2 | CATGAAAGTCTCTGCCGCCC |
| hCCL2Rev / CCL2 | GGGCATTGATTGCATCTGGCTG |
| hCCL20Fw / CCL20 | TTGTGCGTCTCCTCAGTAAAAA |
| hCCL20Rev / CCL20 | TCCAACCCCAGCAAGGTTC |
| hCXCL1Fw / CXCL1 | CTTCCTCCTCCCTTCTGGTC |
| hCXCL1Rev/ CXCL1 | GAAAGCTTGCCTCAATCCTG |
| hCXCL2Fw / CXCL2 | GCTTCCTCCTTCCTTCTGGT |
| hCXCL2Rev / CXCL2 | GGGCAGAAAGCTTGTCTCAA |
| hNFKBIAFw / NFKBIA | AAGGCCAGGTCTCCCTTCAC |
| hNFKBIARev / NFKBIA | CAGCAGCTCACCGAGGAC |
| hTNFAIP2Fw / TNFAIP2 | CTACGCTGGCCGAGATCATT |
| hTNFAIP2Rev / TNFAIP2 | CTCAGGTGGCCTTTGCTGAA |
| hTNFAIP3Fw / TNFAIP3 | GGCCCGGAGAGGTGTTG |
| hTNFAIP3Rev / TNFAIP3 | TCTTCTGGAGTTCTCTCCCGT |
