# Supplementary material for: An Arginine-Rich Motif in the ORF2 capsid protein regulates the hepatitis E virus lifecycle and interactions with the host cell
Source: PLoS Pathog. 2022 Aug 25;18(8):e1010798. doi: 10.1371/journal.ppat.1010798 (PMC9451086; doi:10.1371/journal.ppat.1010798)
Supplement: Attachment — Submitted filename: PLoS-Patho-R2-Comments.pdf [file ppat.1010798.s020.pdf]

Part I - Summary
Please use this section to discuss strengths/weaknesses of study, novelty/significance, general execution and scholarship.

Reviewer #1: The authors added new data in revised manuscript, but the following concerns are still not been addressed.

Reviewer #2: In their manuscript entitled "An Arginine-Rich Motif in the ORF2 Capsid Protein Regulates the Hepatitis E Virus Lifecycle and Interactions with the Host Cell" Hervouet et al. use an HEV cell culture model to elucidate the role of ARM on multiple steps of HEV propagation. Utilizing a series of ORF2 mutants expressed either in the p6 HEV strain or as ORF2 capsid protein they delineate a crucial role of the ARM as functional nuclear localization signal which seems to be important for viral production. With the help of specific inhibitors and co- localization studies, the authors identified a CRM1-dependent egress from the nucleus and 3 important nuclear export signals within the ORF2 protein. The main body of the manuscript focusses on the maturation and addressing of ORF2. The authors found evidence for a role of a furin-dependent secretion pathway in the maturation process. With the generation and evaluation of additional mutants including the signal and/or the encoding sequence of CD4 by state-of the art techniques the authors delineate a tight interplay of the ARM with the respective signal peptide for addressing, topology and egress of the capsid proteins. Bringing this data set together the authors finally delineate a model for the generation of the infectious or non-infectious forms of the HEV particles.
The manuscript is well written, the experiments are appropriate and on a technically high level. Following on the previous reports from the group this manuscript sheds light into the so far under investigated HEV life-cycle and the generation of the different capsid forms. Overall, this manuscript describes several novelties in the field and sets the ground for several future scientific perspectives concerning HEV.

Part II – Major Issues: Key Experiments Required for Acceptance

Reviewer #1:
1) Subcellular localization of ORF2 does not change obviously during infection condition (Fig 1D) compared with electroporation (Fig 1A-C), suggesting that it may be an artifact in electroporation condition due to ORF2 overexpression.
RESPONSE: This reviewer probably did not notice that this point is addressed in the revised manuscript. As mentioned in page 6 line 126 and illustrated in the figure below, infected Huh-7.5 cells displayed ORF2 populations with different N/C ratio. On one hand, a population displayed a nuclear localization which decreased overtime (in red in the figure below). This ORF2 population displayed a same profile than that of electroporated cells (Fig 1A, B, and C). On the other hand, one or two other populations with higher N/C ratio were observed because at 6 days post-infection, a new round of infection occurs and therefore some cells are newly infected and display a N/C ratio as in early time points post-infection.

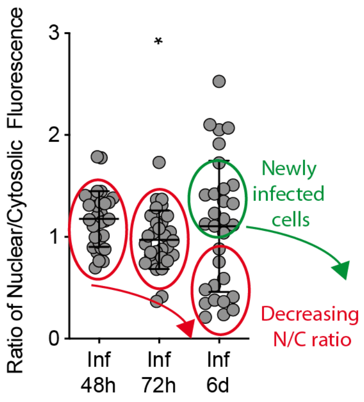

To corroborate this observation, we performed a statistic analysis. We used the gaussian mixture model which looks for a combination of normal distributions and homogeneous clusters. The distribution of nuclear fluorescence intensities at day 6

followed a normal distribution (pvalue of the shapiro test = 0.13, same conclusion at 48 hours and 72 hours post-infection). We used the Rmixmod::mixmodCluster function and tested the distribution of fluorescence intensities from 1 to 4 clusters and the default given models (mixmodGaussianModel). The search of the best model was based on the Stochastic Expectation Maximisation algorithm with random initialization. The results indicated that nuclear fluorescence intensities at day 6 post-infection can be divided into 3 clusters, strengthening the hypothesis that cells were at 3 different stages of infection.
The characteristics of the clusters are given in the table below.

Cluster ID	Mean of nuclear fluorescence intensities	Variance of nuclear fluorescence intensities
1	0.4163	0.0347
2	1.2426	0.0347
3	2.0645	0.0347

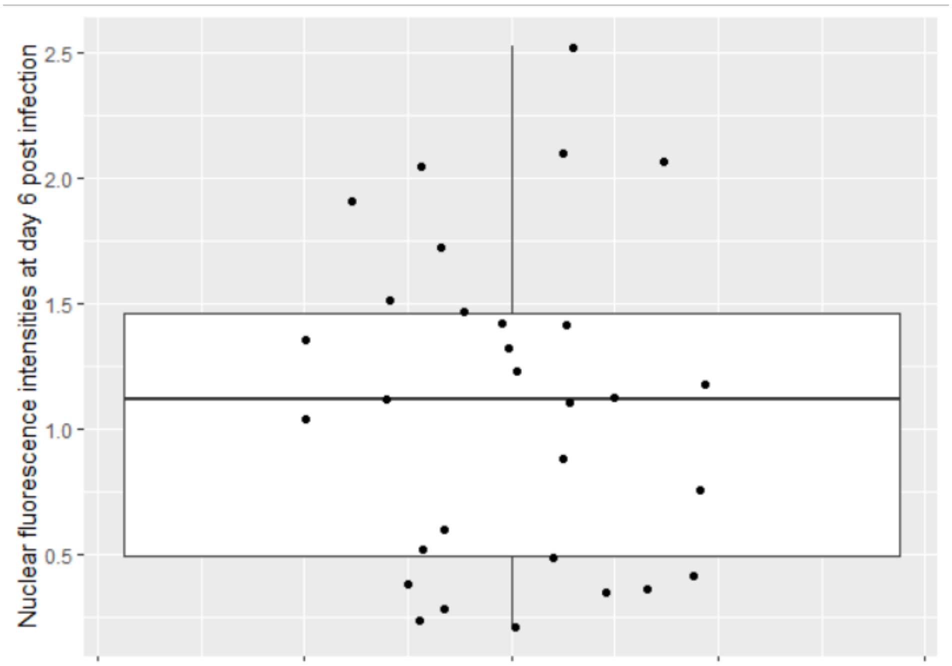

Finally, we performed an additional control in which we treated HEV-p6 infected Huh-7.5 cells with nuclear export inhibitors. As shown in the new S8A Fig, the N/C ratio of ORF2 fluorescence in treated infected cells increased significantly, as was observed for the treated electroporated cells. These results indicate that, in HEV-infected cells, ORF2 is no longer exported from the nucleus as compared to untreated cells, upon treatment with nuclear export inhibitors. Therefore, the ORF2 protein undergoes the same nuclear import/export pathway in electroporated and infected cells.

2) Fig 2D, there are multiple rounds of infection already, so the phenotype could be due the alterations of ORF3. This concern is not been addressed.
According to the result in S7 Fig, the direct conclusion is that deletion of ORF3 don't impair ORF2 nuclear localization. Our question is how to ensure that the fewer infectious particles produced is indeed caused by ARM mutation rather than ORF3 mutation (ORF2 and ORF3 ORFs are largely overlapped)? To answer this question, I think the possible strategies are as follows: (1) When generating ARM mutation to impair ORF2 nuclear localization in the contents of genomic sequence, please make sure the ORF3 amino acids unchanged. (2) utilize

the trans-complementation system to uncouple the expression of ORF2 and ORF3 protein, then investigate the ARM mutation on virus production.

RESPONSE: As mentioned previously, since ORF3 and ORF2 are overlapping, we do agree with the Reviewer 1 that mutations or deletions in ORF2 can cause deleterious effect on expression/functionality of ORF3 protein. Therefore, we monitored ORF3 expression (Fig 2C and 3D) and showed that only ΔSP and ΔSP1 deletions were deleterious for ORF3 whereas ARM mutations did not affect ORF3 expression (Fig 2C). As this protein plays an essential role in particle secretion, ORF3 expression was taken into account for the interpretation of extracellular titers (Fig 2D and 3F).

The ORF3 protein is a small protein with a viroporin activity [1] that has been shown to play an essential role in particle egress but that is not required for particle assembly [2–6]. Therefore, regardless of ORF3 expression/functionality/localization, intracellular infectious titers are representative of HEV particle assembly. As shown in Fig 2D, ARM mutations were totally lethal for assembly of intracellular progeny whereas the ORF3-deficient ΔSP mutant still produced intracellular particles. Therefore, we are convinced that observed phenotypes are not related to ORF3.

3) From the total extract panel in new Fig 2, it is obvious that ORF2 stability is dramatically impaired of ΔSP1, but the authors didn't mention it in response.
RESPONSE: We have taken this comment into consideration and modified the text (see page 8 line 175)

Firstly, in S7 Fig, a WB result is required to show that the deletion of ORF3 is successful. More importantly, this assay (S7 Fig) can't answer our question.
RESPONSE: We demonstrated the absence of ORF3 expression in ΔORF3 construct by immunofluorescence (S6 Fig). However, in accordance with this comment, we performed an additional analysis by WB of different mutants including the ΔORF3 mutant. As shown in the new Fig S7 panel A, the HEV-p6-ΔORF3 mutant does not express the ORF3 protein.

Reviewer #2: This reviewer feels, that all questions have been adequately adressed by the authors in this revision.
RESPONSE: We thank the reviewer for her/his comments and support.

Part III – Minor Issues: Editorial and Data Presentation Modifications
Please use this section for editorial suggestions as well as relatively minor modifications of existing data that would enhance clarity.

Reviewer #1: 1) Please check primers to construct 5R/5A mutation. It seems to be wrong.

RESPONSE: We thank the reviewer for identifying this copy/paste error for the HEV-5 primer (Fw). The HEV-5 sequence corresponds to the first 45 nt, as indicated on the attached delivery note. Following this comment, the S1 table has been modified.

eurofins | Genomics

Rapport de Synthése Oligonucléotide

Page 1/2

M. Maliki Ankavay	N° de Cde:	4022112/11104022112-1	Date de Cde:	03/04/2017	Eurofins Genomics
IBL	Numéro Client:	1118869	N° Labo:	1037	Anzingerstraße 7a
Inserm-U1019 Eq Jean DUBUISSON	Ref Interne Clt:	2017004746/U1019	N° d'Oligos:	14/14	D- 85560 Ebersberg

eurofins | Genomics

Rapport de Synthése Oligonucléotide

Page 2/2

Primers used for the 5R/5A mutant

	N°	Nom de l'Oligo	Séquence (5'->3')	Qté [OD]	Qté [µg]	Qté [nmol]	Concentration [pmol/µl]	Vol. pour 100pmol/µl	Tm [°C]	MW [g/mol]	Taux de GC	Echelle de Synthèse	Purification	Modification	Barcode IDO	Rapport QC
HEV-5 (Fw)	13	NLS3 RRRGRR/AA AGAA-	CCGTCTGGCGCTGCTGC TGGGGCGGCCAGCGGC GGTGCCGGCGGT (45)	10.1 8	316	22,6	-	226	> 75	13961	82,2 %	0.05 µmol	HPSF	-	023443558	MALDI
HEV-6 (Rev)	14	NLS3 RRRGRR/AA AGAA-	ACCGCCGGCACCGCCGC TGGCCGCCCCAGCAGCA GCGCCAGACGG (45)	11.0 1	331	24,1	-	241	> 75	13735	82,2 %	0.05 µmol	HPSF	-	023443559	MALDI

References

1. Ding Q, Heller B, Capuccino JMV, Song B, Nimgaonkar I, Hrebikova G, et al. Hepatitis E virus ORF3 is a functional ion channel required for release of infectious particles. PNAS. 2017; 201614955. doi:10.1073/pnas.1614955114

2. Sari G, Zhu J, Ambardekar C, Yin X, Boonstra A, Feng Z, et al. The viral ORF3 protein is required for hepatitis E virus apical release and efficient growth in polarized hepatocytes and humanized mice. J Virol. 2021; JVI0058521. doi:10.1128/jvi.00585-21

3. Emerson SU, Nguyen HT, Torian U, Burke D, Engle R, Purcell RH. Release of genotype 1 hepatitis E virus from cultured hepatoma and polarized intestinal cells depends on open reading frame 3 protein and requires an intact PXXP motif. Journal of virology. 2010;84: 9059–9069. doi:10.1128/jvi.00593-10

4. Yamada K, Takahashi M, Hoshino Y, Takahashi H, Ichiyama K, Nagashima S, et al. ORF3 protein of hepatitis E virus is essential for virion release from infected cells. The Journal of general virology. 2009;90: 1880–1891. doi:10.1099/vir.0.010561-0

5. Nagashima S, Takahashi M, Jirintai, Tanaka T, Yamada K, Nishizawa T, et al. A PSAP motif in the ORF3 protein of hepatitis E virus is necessary for virion release from infected cells. Journal of General Virology. 2011;92: 269–278. doi:10.1099/vir.0.025791-0

6. Glitscher M, Hildt E. Hepatitis E virus egress and beyond – the manifold roles of the viral ORF3 protein. Cell Microbiol. 2021; e13379. doi:10.1111/cmi.13379
