# Supplementary material for: An Arginine-Rich Motif in the ORF2 capsid protein regulates the hepatitis E virus lifecycle and interactions with the host cell
Source: PLoS Pathog. 2022 Aug 25;18(8):e1010798. doi: 10.1371/journal.ppat.1010798 (PMC9451086; doi:10.1371/journal.ppat.1010798)
Supplement: Attachment — Submitted filename: Reviewers comments PPATHOGENS-D-21-01762-R1.docx [file ppat.1010798.s019.docx]

**Part I - Summary**
Please use this section to discuss strengths/weaknesses of study, novelty/significance, general execution and scholarship.

**Reviewer #1:** The life cycle of HEV is poorly understood due to lack of robust cell culture model. ORF2 is the viral capsid protein, which could package viral genome and mediate viral entry by engagement with the putative cellular receptor. Interestingly, ORF2 has multiple forms and they play different roles in HEV life cycle. In this manuscript, Hervouet et al constructed a panel of ORF2 mutants in Arginine-Rich Motif (ARM) to delineate the function of ARM motif in regulating ORF2s subcellular localization and host antiviral response. ARM regulates the production of different forms of ORF2 and serves as maturation site of glycosylated ORF2. This study could further deep our understanding of HEV life cycle and its interaction with cell host, however, this manuscript is not well organized, not necessarily long and the experiments are not well-control, other explanations or conclusions can be drawn. In addition, the authors performed lots of experiments by electroporation of viral RNA, instead of viral infection, which could potentially lead to some artifacts of the observation. The specific comments could be found as below.

**Reviewer #2:** In their manuscript entitled “An Arginine-Rich Motif in the ORF2 Capsid Protein Regulates the Hepatitis E Virus Lifecycle and Interactions with the Host Cell” Hervouet et al. use an HEV cell culture model to elucidate the role of ARM on multiple steps of HEV propagation.
Utilizing a series of ORF2 mutants expressed either in the p6 HEV strain or as ORF2 capsid protein they delineate a crucial role of the ARM as functional nuclear localization signal which seems to be important for viral production. With the help of specific inhibitors and co-localization studies, the authors identified a CRM1-dependent egress from the nucleus and 3 important nuclear export signals within the ORF2 protein.
The main body of the manuscript focusses on the maturation and addressing of ORF2. The authors found evidence for a role of a furin-dependent secretion pathway in the maturation process. With the generation and evaluation of additional mutants including the signal and/or the encoding sequence of CD4 by state-of the art techniques the authors delineate a tight interplay of the ARM with the respective signal peptide for addressing, topology and egress of the capsid proteins. Bringing this data set together the authors finally delineate a model for the generation of the infectious or non-infectious forms of the HEV particles.

The manuscript is well written, the experiments are appropriate and on a technically high level. Following on the previous reports from the group this manuscript sheds light into the so far under investigated HEV life-cycle and the generation of the different capsid forms.

Overall, this manuscript describes several novelties in the field and sets the ground for several future scientific perspectives concerning HEV.

**Part II – Major Issues: Key Experiments Required for Acceptance**
Please use this section to detail the key new experiments or modifications of existing experiments that should be absolutely required to validate study conclusions.

Generally, there should be no more than 3 such required experiments or major modifications for a "Major Revision" recommendation. If more than 3 experiments are necessary to validate the study conclusions, then you are encouraged to recommend "Reject".

**Reviewer #1:**

1) P6 L114 why the authors choose electroporation of viral RNA genome to observe the ORF2 localization, instead of viral infection? A large amount of viral RNA delivered into the cells by electroporation could produce lots of ORF2, which is not an appropriate model to observe ORF2 localization.

RESPONSE: In the first part of our study, we chose electroporation of viral RNA genome to analyze the ORF2 localization because in the subsequent experiments, we made mutations in the ORF2 sequence that were expected, at least for some of them, to affect ORF2 addressing and thus viral particle assembly, precluding the possibility of characterizing the effect of mutations by infection assays. However, based on this comment and Reviewer 2's comment, we performed additional time-kinetics of nuclear localization of ORF2 in Huh-7.5 cells infected with HEV-p6 particles and PLC3 cells transfected with HEV-Sar55 (gt1) or with another gt3 strain (HEV83-2) (new Fig 1). We found that in PLC3 cells electroporated with HEV-Sar55 RNA (Fig 1B), ORF2 displayed a nuclear localization at early time points p.e. (*i.e.,* 18h) that decreases over time, as observed in PLC3/HEV-p6 cells. In PLC3/HEV83-2 cells (Fig 1C) and HEV-p6 infected Huh-7.5 cells (Fig 1D), the ORF2 expression was delayed but displayed a similar profile. Taken together, these new results indicate that ORF2 nuclear localization is a key feature observed using different ORF2 expression systems, HEV strains or cell types.

2) The authors constructed series of ARM mutants to observe the alterations of ORF2 mutants localization to demonstrate ARM’s NLS function. Also they engineered the mutations accordingly to demonstrate the critical role of ORF2 nuclei localization in HEV life cycle (Fig 2A-D). It has been well known that ORF2 is also responsible to package viral genome to form infectious particles, and it is very possible that the ARM mutation impaired ORF2 function to package viral genome, which is not related to its NLS function. I suspect these mutations affect ORF2 fold or interaction with genomic RNA to cause the phenotype.

RESPONSE: We thank this Reviewer for giving us the opportunity to address this important point. Indeed, although the mechanisms driving HEV assembly are poorly understood, it is believed that encapsidation is launched by an interaction between the 5’ end of the genomic RNA and positively charged residues within the first 110 amino acid of ORF2 (Surjit *et al.*, 2004), followed by a spontaneous ORF2 oligomerization. However, it is difficult to test the effect of ARM mutations on the ORF2/RNA interaction since ORF2 mutations lead viral components to be in different cell compartments and thus distant from each other. In addition, very recently, by AlphaFold2 modeling of the ORF2 N-terminus, we found that the ORF2 ARM is exposed on the surface of viral particle whereas the C-terminal end of the N-terminus is likely orientated toward the interior cavity of particles and thus interacting with viral genome. These results are part of another study on characterization of viral particles and factories that is currently under revision.

However, following Reviewer 1's suggestion to probe the effect of ARM mutation on ORF2 folding, we performed immunoprecipitation experiments with conformational anti-ORF2 antibodies (2E2 and 4B2) on total cellular extract. As shown in the new Fig 2, we found that all ORF2 mutants were recognized similarly by antibodies, as compared to the inputs, indicating that none of the mutations drastically affect ORF2 folding.

3) In addition, the authors also need to check the total ORF2 amount in Fig 2C, excluding the possibility that the mutations in ARM affect ORF2 stability.

RESPONSE: We thank again this Reviewer for giving us the opportunity to address this important point. An anti-ORF2 WB on total cellular extract has been added in the new Fig 2 with the immunoprecipitation experiments with conformational anti-ORF2 antibodies. This new gel indicates that none of the mutations drastically affect ORF2 stability and only the SP deletion (ΔSP construct) led to a slightly less expressed and/or less stable ORF2 protein.

The cellular markers in the WB were required to make sure the quality of isolation of each fraction. For example, there is not nuclei maker (histone protein) in the nuclei extract, which is very important.

RESPONSE: This concern has already been addressed. Indeed, Tubulin, Calnexin (CNX) and SP1 were used to control the quality of cytosolic, membrane and nuclear fractions, respectively. Detection of SP1 transcription factor (used in Figs 2 and 3) or Lamin (used in Figs 5 and 6) was preconized by the manufacturer (Thermo Scientific) to control nuclear fraction.

4) Data presented in Figure 2D show the ORF2 mutants produce less infectious viral particles than WT. As the ORF2 and ORF3 are overlapped, in ORF2 mutants, the amino acid of ORF3 also changed, therefore, more data should be presented to exclude the possibility that this phenotype is caused by ORF3 mutation.

RESPONSE: We agree with the Reviewer 1 that mutations or deletions in ORF2 can cause a defect in ORF3. Therefore, in our study, we monitored the expression of ORF3 (Fig 2C and 3D). These analyses showed that only ΔSP and ΔSP1 deletions were deleterious for ORF3 expression (Fig 2C). Since this protein plays an essential role in particle secretion, ORF3 expression was taken into account for the interpretation of extracellular titers (Fig 2D and 3F).

Of note, a time-kinetics of ORF2 localization in cells expressing the ΔORF3 mutant showed that ORF2 nuclear translocation was similar to that of wt (S7 Fig), indicating that the phenotypes observed for the nuclear localization of ORF2 mutants cannot be attributed to ORF3.

5) The authors used multiple inhibitors to block the interested pathway and then analyze the phenotype (Fig 3 and Fig 4), and always have the concerns about the specificity of the phenotype in the manuscript. More solid data should be used to support the conclusions.

RESPONSE: Indeed, in order to analyze the nuclear export process of the ORF2 protein, we used two selective inhibitors of nuclear export (SINE), namely Leptomycin B which is an irreversible inhibitor of CRM1 and Verdinexor a reversible inhibitor of CRM1. The specificity of these molecules has been well characterized (Gravina *et al.*, 2014). Furthermore, cells treated with SINE and cells expressing NES ORF2 mutants displayed the same nuclear accumulation of ORF2 (Fig 3A and 3C), indicating that the phenotype observed in treated cells was not related to a pleiotropic effect of the drugs.

Of note, we performed additional SINE treatments of PLC3 cells electroporated with the HEV83-2 strain and demonstrated the same ORF2 nuclear accumulation of ORF2 upon treatment (S8A Fig), indicating that this phenotype is not strain specific.

Another approach to confirm the importance of CRM1 in the HEV lifecycle would be to knockout its expression. However, since CRM1 is the predominant carrier for export of proteins and RNA from the nucleus into the cytoplasm, its knockout in PLC3/HEV-p6 cells is toxic.

To strengthen our findings on the importance of CRM1 in the HEV lifecycle, we set up proximity ligation assays (PLA) to probe ORF2/CRM1 interaction. As shown in S8C Fig, ORF2 and CRM1 likely interact with each other. In addition, mutation of NES12 motif reduces this interaction. Together, these data confirm that ORF2 nuclear export is a CRM1-dependent process.

In Fig 4, in order to analyze the maturation process of the glycosylated ORF2 proteins, we used three different furin inhibitors and related proprotein convertases. We believe that these experiments were well controlled. Indeed, they were controlled by detecting intracellular ORF2 and tubulin to assess ORF2 expression/stability and cell toxicity/protein loading, respectively. We also added an internal control of inhibitor efficacy by probing cleavage of cellular αV-pro-integrin, a substrate of intracellular Furin. Nevertheless, we further supported our results by transfecting siRNAs directed against Furin and confirmed that the maturation of glycosylated ORF2 protein is mediated by Furin (Fig 4G).

**Reviewer #2:**

I have the following major comments.
Major:
All experiments have been performed with the (sub-)genotype Kernow p6. As the authors also stated in their introduction, several other (sub-)genotypes exist with marked differences in their clinical presentation. Therefore, this study would largely benefit from expanding the range of viruses used. In this regard it would be helpful to

I) show by phylogenetic analysis of for example published strains the abundance of ARM as well as NES

RESPONSE: We thank this Reviewer for giving us the opportunity to address this important point. We performed an alignment of ORF2-p6 with 50 ORF2 sequences from gt1, gt2, gt3 and gt4. As now shown in S1 and S9-S11 Figs, the conservation of ARM, NES9, NES10 and NES12 motifs are now well illustrated.

II) show a similar nuclear pattern for a different GT3 subgenotype as well as another genotype (the published data so far only investigated GT3 infections).

RESPONSE: Based on this comment and Reviewer 1's comment, we performed additional kinetic analyses of nuclear localization of ORF2 in PLC3 cells transfected with HEV-Sar55 (gt1) or with another gt3 strain (HEV83-2) and Huh-7.5 cells infected with HEV-p6 particles (new Fig 1). We found that in PLC3 cells electroporated with HEV-Sar55 RNA (Fig 1B), ORF2 displayed a nuclear localization at early time points p.e. (*i.e.,* 18h) that decreases over time, as observed in PLC3/HEV-p6 cells. In PLC3/HEV83-2 cells (Fig 1C) and HEV-p6 infected Huh-7.5 cells (Fig 1D), the ORF2 expression was delayed but displayed a similar profile. Moreover, when treated with selective inhibitors of nuclear export, PLC3/HEV83-2 cells showed a strong ORF2 nuclear accumulation (S8A Fig). Taken together, these new results indicate that ORF2 nuclear localization is a key feature observed using different ORF2 expression systems, HEV strains or cell types.

The main body of the manuscript is focussing on virus addressing. Some parts are comparable little studied and therefore do not allow as strong statements as have been made in the manuscript. This in particular is the case for the following:
I) Page 7, Lines 140-143: “In addition, high molecular weight forms of ORF2intra in the soluble fraction as well as an increase of ORF2g/c secretion were observed (Fig2C), suggesting a higher translocation into the secretory pathway associated to an improved functionality of ORF2 SP”
This statement seems only to be true for 3R/3A and 5R/5A, not for the 2R/2A construct. The authors should either repeat the experiments to identify significant results or be gentle with the description.

RESPONSE: We agree with Reviewer 2 that in WB analyses the 2R/2A mutant looks less translocated into the ER lumen. We repeated the experiments and found the same profile. We therefore modified the text as suggested (see page 7, lines 149-156).

Of note, the 2R/2A mutant was more secreted than wt (Fig 2C, supernatant) and Pearson's correlation coefficients calculated to evaluate the co-distribution of ORF2/GM130 (Fig S3) showed a significant effect of the 2R/2A mutations on translocation of ORF2 in the secretory pathway.

II) Page 7, Lines 166-168:”Intracellular titers were also lowered in SP mutants (Fig2D), indicating that the ORF2 SP likely plays an important role in the assembly of infectious particles.” The intracellular HEV RNA is already significantly lower for the SP mutants intracellularly and therefore argue for a reduced replication.

RESPONSE: The text has been modified in accordance with this comment (see page 8, line 181).

Overall, this figure would benefit from a positive control (such as ribavirin) and possibly a time-course to ensure a decent measuring window.

RESPONSE: Following this comment, we have added a supplementary figure (S4 Fig) in which controls of infectious and RNA titers are presented. PLC3/HEV-p6 cells were treated with 20 μM of Sofosbuvir. We also used PLC3 cells electroporated with a replication-deficient GAD p6 genome

III) Page 8, Lines 177-179 “Interestingly, ORF2 and Importin-α1 co-localized in the nucleus of infected cells with a Pearson correlation coefficient (PCC) of 0.670, and the mutation of arginine residues drastically reduced this colocalization. Taken together, our results indicate that ORF2 colocalizes with Importin-α1 thanks to its ARM that serves as a functional NLS.”
This seems to be only a moderate co-localization. More work needs to be done to support this assumption such as performing IP experiments to reveal direct interaction.

RESPONSE: In order to address this comment, we tried to perform co-immunoprecipitations experiments. However, we were unable to identify the best lysis conditions that preserved specific protein-protein interactions without too much background. Therefore, we set up proximity ligation assays to probe ORF2/Importin interaction. As indicated in our results shown in a new Fig. 2E, ORF2 and Importin-α1 interact with each other.

IV) Page 13, Lines 203-207: “Altogether our results suggest that the ORF2 ARM, which notably regulates ORF2 nuclear translocation, is a pivotal viral determinant for the modulation of host pathways and, especially, genes of the NF-κB-induced signalling upon infection. Further studies will be required to define precisely the impact of this HEV-driven host regulation on immune cell responses.”
Although it seems to be an interesting observation, I agree that the displayed data is too preliminary and should be left out or expanded significantly (using different genotypes of HEV, display all identified hits).

RESPONSE: We agree with the reviewer that the displayed data might seem very preliminary. Therefore, we mentioned several times in the manuscript that further analyses would be needed to define precisely the impact of this HEV-driven host regulation on immune cell responses. Due to financial constraints, we were unable to extend these analyses to other strains. However, we believe that our data do illustrate the host response to HEV infection. Similarly, the comparison of p6-wt and p6-5R/5A demonstrates the importance of the ARM in this process. As mentioned previously, the ARM is well conserved in different HEV strains as well as the nuclear translocation of ORF2, showing that the nuclear translocation of ORF2 protein is important for the HEV lifecycle.

Following this comment, we moved the transcriptomic analyses of Fig 2 to S6 Fig and modified the title of the paragraph (page 9, line 203).

Additionally, the time-course of viral replication might be significantly affected depending on whether the construct enters the nucleus or not. Therefore, the analysis at one specific time-point (18h) is difficult to compare. Additionally, mutant delORF3 has not been investigated in the previous experiments in terms of translocation to the nucleus.

RESPONSE: To address this concern, we performed kinetic analyses of ORF2 localization in cells expressing different mutants including the 5R/5A and ΔORF3 mutants (S2 and S7 Fig). These new results showed that 18 hours p.e. was a good time-point to compare gene modulation related to ORF2 nuclear translocation. Moreover, our new results demonstrated that in the absence of ORF3 expression, ORF2 nuclear translocation was similar to that of wt (S7 Fig), indicating that ORF2 nuclear translocation is an ORF3-independent process and the ΔORF3 mutant is a relevant control for studying gene expression modulation by ORF2 nuclear translocation.

V) Fig 7/8. Have the authors analysed an additional SP from a different source in terms of its phenotype?

RESPONSE: We are not sure we fully understand the reviewer's concern. As described in the manuscript, we performed an extended analysis of ORF2 addressing that is mediated by a fine interplay between its signal peptide and the ARM. We generated a series of chimeric proteins in which we exchanged the ORF2 signal peptide with that of CD4, which is a functional model signal peptide. To strengthen our findings, we also generated a series of chimeric proteins in which we exchanged the CD4 signal peptide with that of ORF2. Characterization of these series of chimeric proteins allowed us to demonstrate that a specific interplay between ORF2 signal peptide and ARM controls the fate of ORF2 protein.

Following this comment, we added the schematic representation of ORF2 and CD4 full-length proteins in Fig 7 and 8.

**Part III – Minor Issues: Editorial and Data Presentation Modifications**
Please use this section for editorial suggestions as well as relatively minor modifications of existing data that would enhance clarity.

**Reviewer #1:**

1) Fig 1, it is required to present the nuclei staining results.

RESPONSE: The Fig 1 has been modified following this comment

2) Fig 2D needs RdRp inactive (GAD) control.

RESPONSE: Following this comment, we have added a supplementary figure (S4 Fig) in which controls for infectious and RNA titers are shown. PLC3/HEV-p6 cells were treated with 20 μM of Sofosbuvir. As requested by the reviewer, we have also added the results of PLC3 cells electroporated with a replication-deficient GAD p6 genome

3) P9 L187 whether overexpression of ORF2 can cause similar results on NF-kB-related signaling.

RESPONSE: We did not analyze the effect of ORF2 overexpression on NF-kB related pathway.

4) P10 L230 The phenotype is not consistent among these three mutants, suggesting that there is nothing to do with nuclear localization.

RESPONSE: We believe that there are no major discrepancies between NES mutants. The NES12 mutant displayed lower nuclear localization as compared to NES9 and NES10 mutant but displayed a highly significant nuclear accumulation, as compared to wt. However, following Reviewer 1's suggestion to probe the effect of ARM mutations on ORF2 folding (see above, Part II, Rev 1, Concern 2), we have also probed the effect of NES mutations on ORF2 folding by immunoprecipitation experiments with conformational anti-ORF2 antibodies (2E2 and 4B2) on total cellular extract. As shown in the new Fig 3, we found that NES9 and NES10 mutants were no longer recognized by 2E2 and 4B2 Ab whereas NES12 was recognized similarly by antibodies, as compared to wt and inputs (new Fig 3D**,** IP2E2 and IP 4B2), suggesting that NES9 and NES10 mutants were misfolded whereas NES12 mutant was properly folded. These results indicate that NES9 and NES10 motifs are functional NES but their mutation affect ORF2 protein folding and thus particle assembly.

Based on these new results, we have modulated our conclusions and modified the text.

Regarding the NES12 mutant, we performed an additional time-kinetics of ORF2 localization and again found a significant nuclear accumulation of this mutant, as compared to wt (S7 Fig). Therefore, the reduced infectious particle assembly of the NES12 mutant is likely due solely to its differential subcellular localization and not to a defect in viral replication or ORF2 folding.

Based on these new results, we have modulated our conclusions and modified the text.

5) P13 L287-L289 The localization of ORF2 is also dynamic like infection condition? If not, OE system cannot reflect true functional ORF2 in infection condition.

RESPONSE: We used a heterologous expression system for ORF2 to study its addressing mechanisms and not to study its localization dynamics. Our analyses showed that ORF2 wt or mutants had similar phenotypes in infectious and heterologous systems. They exhibited similar subcellular distribution, secretion, membrane association and nuclear localization, indicating that heterologous system was a relevant system for studying ORF2 addressing mechanisms. Moreover, since we generated chimeric ORF2/CD4 proteins, we could not use an infectious system.

6) Figure 2C. Tubulin of ΔSP1 is much less than the others, indicating the quality of ΔSP1 soluble extract is not good.

RESPONSE: Following this comment, we performed a new migration and Tubulin blotting of samples. The gel of Fig 2C has been replaced by the new one. As demonstrated by this new gel, the amounts of Tubulin were equal in all samples.

7) Figure 4A and 4D-F. At What time points post-electroporation are the cells treated with inhibitors? Some related descriptions should be added to the legend.

RESPONSE: In Fig 4, inhibitor treatment and siRNA transfection were performed on cells at 7 days post-electroporation.

Following this comment, the legend of Fig 4 has been modified.

**Reviewer #2:** Overall, the study would benefit from additional controls and more detailed analysis in particular in the first part.

1) Supplement Figure 6: Please provide data with addition of a positive/cytotoxic control.

RESPONSE: Following this comment, we added a dose-response curve of PLC3 cells treated with concentrations superior to 20 µM of Gossypol that are cytotoxic (new S13 Fig).

2) Page 7 Lines 151-155: he PSG/3R mutant showed a marked nuclear localization (Figs2Band 2C, Nuclear extract) but was impaired in ORF2g/c secretion (Fig2C, Supernatant), as observed for the SP deletion mutants, indicating that the addition of arginine residues strengthens the NLS function of ARM but inhibits the functionality of ORF2 SP.
To underline this point it would be interesting to perform an additional time-kinetic (such as in Fig1) to understand and learn where the ORF2 dose eventually reside and if it is retained in the nucleus for a longer period of time.

RESPONSE: Following this comment, we performed additional time-kinetics of nuclear translocation of five mutants/constructs. As requested, we further analyzed the PSG/3R mutant and the ΔSP construct (new S2 Fig) but we also analyzed the 5R/5A (new S2 Fig), ΔORF3 and NES12 mutants (new S7 Fig). As shown in the new S2 Fig, the ΔSP construct and especially the PSG/3R mutant highly translocate, accumulate and stay longer in the nucleus.

3) Page 14, Lines 231-234, “Of note, intracellular replication was not altered by NES mutations (Fig3F), indicating that the loss of infectious particle assembly is due to the differential subcellular localization of the mutants and not to a replication defect.”
This seems counterintuitive. Please provide appropriate replication inhibitor control of the assay. Additionally, this would also benefit from a time-kinetic analysis.

RESPONSE: As mentioned previously, we have added a supplementary figure (S4 Fig) in which controls for infectious and RNA titers are shown, and we performed additional time-kinetics of PLC3 cells expressing mutants including the NES12 mutant (S2 and S7 Figs).

4) In line 114 page 6: Titel: “phase of infection”: This is not correct, as the cells were transfected. Therefore, the term replication would be more appropiate.

RESPONSE: The text has been modified following this comment

5) Fig 5C convincingly shows that nuclear ORF2 is present in the WT construct. However, for Fig5B this is difficult to judge by eye.

RESPONSE: We assume that the reviewer is referring to Fig 5A (IF) and not to Fig 5B (Ratio N/C).

Following this comment, we selected another image for ORF2wt (Fig 5A).

In Fig. 2C the nuclear signal is displayed as nuclear fluorescent intensity. However, in the following figures this is delineated as nuclear/cyto intensitiy ratio, which makes a comparison difficult. Please harmonize the display of these results.

RESPONSE: We assume that the reviewer is referring to Fig 2B (IF and quantification of nuclear fluorescence) and not to Fig 2C (WB).

Following this comment, we calculated the ratio of nuclear/cytosolic fluorescence for each mutant displayed in Fig 2. We thank the Reviewer for giving us the opportunity to address this point because in this way the effect of ARM mutations on nuclear localization is better illustrated.

**References:**

Gravina GL, Senapedis W, McCauley D, Baloglu E, Shacham S, and Festuccia C. Nucleo-cytoplasmic transport as a therapeutic target of cancer. 2014 Journal of Hematology & Oncology, 7:85

Surjit M, Jameel S, and Lal SK. The ORF2 Protein of Hepatitis E Virus Binds the 5' Region of Viral RNA. 2004. J. Virol, 78, 320-328

PLOS authors have the option to publish the peer review history of their article ([what does this mean?](https://journals.plos.org/plospathogens/s/editorial-and-peer-review-process#loc-peer-review-history)). If published, this will include your full peer review and any attached files.


If you choose “no”, your identity will remain anonymous but your review may still be made public.


**Do you want your identity to be public for this peer review?** For information about this choice, including consent withdrawal, please see our [Privacy Policy](https://www.plos.org/privacy-policy).

Reviewer #1: No

Reviewer #2: No

Figure Files:

While revising your submission, please upload your figure files to the Preflight Analysis and Conversion Engine (PACE) digital diagnostic tool, [https://pacev2.apexcovantage.com](https://pacev2.apexcovantage.com/). PACE helps ensure that figures meet PLOS requirements. To use PACE, you must first register as a user. Then, login and navigate to the UPLOAD tab, where you will find detailed instructions on how to use the tool. If you encounter any issues or have any questions when using PACE, please email us at [figures@plos.org](mailto:figures@plos.org).

Data Requirements:

Please note that, as a condition of publication, PLOS' data policy requires that you make available all data used to draw the conclusions outlined in your manuscript. Data must be deposited in an appropriate repository, included within the body of the manuscript, or uploaded as supporting information. This includes all numerical values that were used to generate graphs, histograms etc.. For an example see here on PLOS Biology: <http://www.plosbiology.org/article/info%3Adoi%2F10.1371%2Fjournal.pbio.1001908#s5>.

Reproducibility:

To enhance the reproducibility of your results, we recommend that you deposit your laboratory protocols in protocols.io, where a protocol can be assigned its own identifier (DOI) such that it can be cited independently in the future. Additionally, PLOS ONE offers an option to publish peer-reviewed clinical study protocols. Read more information on sharing protocols at <https://plos.org/protocols?utm_medium=editorial-email&utm_source=authorletters&utm_campaign=protocols>
